# Integrated multi-omics reveals gut microbiota–metabolic mechanisms underlying the neuroprotective effects of Xijiao Dihuang Decoction against cerebral ischemia–reperfusion injury

**DOI:** 10.3389/fmicb.2026.1851608

**Published:** 2026-10-15

**Authors:** Yu-yan Long, Yu-jing Feng, Kai-ni Mao, Zhi-guang Song, Yu-chen Liu, Si-yuan Chen, Cai-xing Zheng, Yi-di Zeng, Shu-ting Yin, Si-si Dai, Hua Li, Wang-hua Liu

**Affiliations:** 1 Key Laboratory of TCM Diagnostics of Hunan Province, Hunan University of Chinese Medicine, Changsha, Hunan, China; 2 Hunan Engineering Technology Research Center for Medicinal and Functional Food, Changsha, Hunan, China; 3 Key Laboratory of TCM Heart and Lung Syndrome Differentiation & Medicated Diet and Dietotherapy, Changsha, Hunan, China; 4 Laboratory Animal Center, Hunan University of Chinese Medicine, Changsha, Hunan, China

**Keywords:** 16S rDNA sequencing, cerebral ischemia–reperfusion injury, gut microbiota, neuroprotective effects, untargeted metabolomics, Xijiao Dihuang Decoction

## Abstract

**Background:**

Cerebral ischemia–reperfusion injury (CIRI) remains a major barrier to functional recovery after reperfusion therapy for ischemic stroke. Increasing evidence indicates that gut microbiota dysbiosis and metabolic disturbance are closely involved in post-stroke neuroinflammation and secondary brain injury. Xijiao Dihuang Decoction (XDD), a classical traditional Chinese medicine formula, has shown protective effects in cerebrovascular disorders; however, whether its effects against CIRI are associated with coordinated regulation of the gut microbiota and host metabolism remains unclear.

**Methods:**

Circulating XDD-derived components were characterized by ultra-high-performance liquid chromatography–tandem mass spectrometry (UHPLC–MS/MS). Network pharmacology and molecular docking were used to predict potential targets and signaling pathways associated with CIRI. A middle cerebral artery occlusion/reperfusion (MCAO/R) rat model was established, and rats were treated with low-, medium-, or high-dose XDD for 7 days, with edaravone as a positive control. Neurological deficits, cerebral infarct volume, hemorheological parameters, histopathological changes, and serum inflammatory cytokines were assessed. Cecal microbiota composition was analyzed by 16S rDNA sequencing, and cecal metabolic profiles were investigated using untargeted LC–MS metabolomics. Multi-omics integration and Spearman correlation analysis were performed to explore associations between differentially abundant bacterial genera and characteristic metabolites.

**Results:**

A total of 54 XDD-derived circulating components were identified. Network pharmacology analysis indicated that the putative targets of XDD were mainly enriched in pathways related to inflammation, lipid and energy metabolism, and regulated cell death, with AKT1 and NLRP3 were predicted as potential hub targets. In MCAO/R rats, XDD improved neurological deficits, reduced cerebral infarct volume, alleviated hemorheological abnormalities, and attenuated neuronal injury. XDD also reduced serum IL-6 levels and lowered IL-1β levels mainly at medium and high doses. In addition, XDD partially restored gut microbial diversity and remodeled CIRI-associated microbial dysbiosis. Metabolomics analysis revealed marked metabolic disturbances after MCAO/R, and XDD reversed part of these alterations, particularly those related to lipid metabolism. Integrated analysis further linked specific bacterial genera with lipid mediators and fatty acid-related metabolites, indicating coordinated remodeling of the gut microbiota and host metabolism.

**Conclusion:**

XDD exerts neuroprotective effects against CIRI through multi-target and multi-pathway actions. Its therapeutic effects may be associated with coordinated regulation of gut microbial homeostasis, metabolic reprogramming, and inflammatory/cell death signaling. These findings provide new evidence supporting the potential application of XDD in ischemic stroke and offer insight into microbiota-related mechanisms underlying its neuroprotective effects.

## 1 Introduction

Stroke remains one of the leading causes of death and long-term disability worldwide, with ischemic stroke accounting for the majority of cases (Feigin et al., 2025; Tu et al., 2023). Timely restoration of cerebral blood flow by intravenous thrombolysis or endovascular therapy is the cornerstone of ischemic stroke treatment. However, the clinical benefits of reperfusion therapy are limited by narrow therapeutic windows and the risk of secondary cerebral ischemia–reperfusion injury (CIRI) (Feske, 2021; Hilkens et al., 2024). CIRI is characterized by a complex pathological cascade involving oxidative stress, neuroinflammation, metabolic dysfunction, and regulated cell death, which together aggravate neuronal injury and impair neurological recovery (Zhao Y. et al., 2022). Therefore, identifying safe and effective therapeutic strategies that target multiple pathological processes remains an important challenge in ischemic stroke management.

Traditional Chinese medicine (TCM), with its multi-component and multi-target characteristics, has attracted increasing attention as a complementary therapeutic strategy for ischemic stroke (Liu et al., 2018; Zhu et al., 2022). According to TCM theory, the coexistence of heat and blood stasis is closely associated with the pathological progression of CIRI. Xijiao Dihuang Decoction (XDD), a classical formula first recorded in *Beiji Qianjin Yaofang*, consists of Cornu Bubali, Rehmanniae Radix, Paeoniae Radix Rubra, and Moutan Cortex. Traditionally, XDD has been used to clear heat, cool the blood, detoxify, and resolve blood stasis, and has been prescribed for apoplexy, febrile coma, and blood-heat-related disorders.

Modern pharmacological studies have provided evidence supporting the protective effects of XDD in cerebrovascular diseases. Clinical and experimental studies suggest that XDD can improve blood-heat and blood-stasis-related conditions and attenuate ischemic brain injury, possibly through the regulation of inflammation, neurogenesis, angiogenesis, and TLR4–MyD88/NF-κB signaling (Chen et al., 2022; Fei et al., 2018; Zhang et al., 2017). However, most existing studies have focused on individual herbs, isolated compounds, or specific inflammatory pathways. The systematic mechanisms by which XDD, as an integrated formula, exerts neuroprotective effects against CIRI remain insufficiently understood.

Accumulating evidence indicates that the gut microbiota plays an important role in stroke pathogenesis and recovery through the microbiota–gut–brain axis (Durgan et al., 2019; Peh et al., 2022; Zhang et al., 2023). Gut microbial dysbiosis can influence intestinal barrier integrity, systemic inflammation, immune responses, and host metabolic homeostasis, thereby contributing to secondary brain injury after stroke. Meanwhile, herbal medicines have been shown to regulate gut microbial composition and microbial metabolite profiles, providing a potential mechanistic bridge between traditional therapeutic effects and modern biological pathways (Che et al., 2022; Feng et al., 2018). Nevertheless, whether the neuroprotective effects of XDD against CIRI are associated with coordinated regulation of gut microbiota and host metabolites remains unclear.

Based on these considerations, we hypothesized that XDD may protect against CIRI through a coordinated “XDD formula–gut microbiota–metabolites–neuroprotection” mechanism. To test this hypothesis, the present study integrated serum pharmacochemistry, network pharmacology, molecular docking, 16S rDNA sequencing, and untargeted metabolomics in an MCAO/R rat model. Circulating XDD-derived components were first characterized by UHPLC–MS/MS, and potential targets and pathways related to CIRI were predicted and validated in silico. The neuroprotective effects of XDD were then evaluated by neurological, histopathological, hemorheological, and inflammatory assessments. Finally, gut microbial composition and cecal metabolic profiles were analyzed to explore the association between XDD-mediated microbiota remodeling, metabolic reprogramming, and neuroprotection. This study provides an integrated multi-omics perspective on the mechanisms underlying the protective effects of XDD against CIRI.

## 2 Materials and methods

### 2.1 Herbal preparation

XDD consists of Cornu Bubali (from artificially raised buffalo), Rehmanniae Radix, Paeoniae Radix, and Moutan Cortex, with detailed composition and proportions provided in Table 1. The botanical identities were confirmed as *Rehmannia glutinosa* (for Rehmanniae Radix), *Paeonia lactiflora* (for Paeoniae Radix Rubra), and *Paeonia suffruticosa* (for Moutan Cortex), with reference to the Chinese Pharmacopoeia and the World Flora Online database (accessed on 30 January 2026).

**Table 1:** Composition of XDD.

Latin name	Chinese name	Medicinal part	Dosage(g)
*Cornu Bubali*	Shuiniujiao	Cornu	30
*Rehmannia glutinosa*	Shengdihuang	tuberous root	24
*Paeonia lactiflora*	Chishao	Dried root	12
*Paeonia suffruticosa*	Mudanpi	Dried root bark	9

XDD was prepared following the original prescription described in *Beiji Qianjin Yaofang*, adhering to the classical formulation ratio and referencing previously published studies (Lu et al., 2020;Zhou G. et al., 2024). All herbal materials were purchased from the First Affiliated Hospital of Hunan University of Chinese Medicine. The batch numbers of Cornu Bubali, Rehmanniae Radix, Paeoniae Radix Rubra, and Moutan Cortex were 2,411,263, XZ25031204, XZ25021903, and CK25031005, respectively. The sources, macroscopic characteristics, and properties of each herb were authenticated by Professor Wanghua Liu to ensure authenticity and quality, in compliance with the Chinese Pharmacopoeia (2020 edition). Voucher specimens were kept at Key Laboratory for TCM Diagnostic Technology, Hunan Province.

UHPLC–MS/MS analysis confirmed the absence of known toxic compounds in the XDD preparation. During the observation period, no drug-related adverse effects were found in the high-dose treatment group which supports that administration of XDD is safe.

All herbal materials were processed into a concentrated decoction using a traditional aqueous extraction method. The mixed herbs were steeped in distilled water for 30 min prior to extraction, followed by a two-step decoction procedure. In the initial step, the materials were subjected to reflux with five volumes of distilled water for 1 h. Subsequently, in the second step, the residue was re-extracted using three volumes of distilled water under the same experimental conditions. To reach an end concentration at 1 g raw herbal material per ml, the filtrates would be concentrated by rotary evaporator under reduced pressure. Decoction is kept dark and refrigerated at 4 °C, equilibrating to room temperature in a 37 °C water bath before giving.

### 2.2 Major reagents

Methanol (CNW Technologies, CAS No. 67-56-1), acetonitrile (CNW Technologies, CAS No. 75-05-8), and ammonium acetate (Sigma-Aldrich, CAS No. 631-61-8) were used for chromatographic analysis. 2,3,5-Triphenyltetrazolium chloride (TTC) staining solution was obtained from Sigma-Aldrich (T8877). Enzyme-linked immunosorbent assay (ELISA) kits for rat interleukin-1β (IL-1β) and interleukin-6 (IL-6) were purchased from Enzyme Immunoassay Biotechnology (MM-0047R1/2 and MM-0190R1/2, respectively).

Anhydrous ethanol (10009218), xylene (10023418), hydrochloric acid (10011018), ammonia solution (10002108), and neutral balsam mounting medium (10004160) were purchased from Sinopharm Chemical Reagent Co., Ltd. Hematoxylin–eosin (H&E) staining solution (B1003), toluidine blue staining solution (B1007), and glacial acetic acid (B1131) were obtained from Wuhan BQD Biotechnology Co., Ltd.

### 2.3 Instruments and equipment

Chemical analysis was done by an ultra-high performance liquid chromatography system (UHPLC) with a high-resolution Orbitrap Exploris 120 mass spectrometer (Thermo Fisher Scientific). Histological and image analyses were carried out using a Nikon ECLIPSE E100 upright optical microscope connected to a Nikon DS-U3 imaging system (Nikon, Japan).

Additional equipment used included the Tissumizer (Omni International, Omni TH), freeze dryer (Labconco FreeZone 2.5, Labconco), ultrasonic cleaner (Elma Transsonic T460, Elma), embedding center (Thermo Shandon, Thermo Fisher), rotary microtome (Leica RM2125 RTS, Leica, Shanghai), CO₂ incubator (MCO-170AICUVL-PA, Panasonic), and an SMR60047 microplate reader (USCNK).

### 2.4 Animals

A total of 100 male specific pathogen-free (SPF) Sprague–Dawley (SD) rats weighing 260 ± 30 g were obtained from Hunan Hujingda Laboratory Animal Co., Ltd. (animal certificate No. 430727251101083423). Six rats were used for the preparation of XDD-containing serum for pharmacochemical analysis, whereas the remaining 94 rats entered the *in vivo* efficacy experiment.

All animal procedures were conducted in accordance with the NIH Guide for the Care and Use of Laboratory Animals and relevant Chinese regulations and were approved by the Institutional Animal Care and Use Committee (approval No. SLBH-202502150001). Rats were housed under controlled conditions at 18 °C–25 °C and 35%–55% relative humidity under a 12-h light/dark cycle.

### 2.5 Analysis of XDD-derived circulating components

#### 2.5.1 Collection of XDD-containing serum

Six SD rats were randomly divided into a sham group and an XDD-containing serum group. The dose for the XDD group was determined according to the body surface area conversion between humans and rats. Rats in the XDD-containing serum group were administered XDD by gavage at 10 times the maximum human-equivalent dose (3.8 g/kg) once daily for 7 consecutive days, whereas rats in the sham group received an equal volume of normal saline. After the last administration, rats were anesthetized by intraperitoneal injection of 2% sodium pentobarbital (40 mg/kg). After deep anesthesia was confirmed, whole blood was collected aseptically from the abdominal aorta. Blood samples were allowed to clot at room temperature for 2 h and then centrifuged at 3,500 rpm for 15 min. The collected serum was heat-inactivated at 56 °C for 30 min and stored at −80 °C until analysis.

#### 2.5.2 Sample pretreatment

Preparation of serum samples: For serum sample preparation, 200 μL of serum was mixed with 20 μL of 2 mol/L hydrochloric acid and 780 μL of acetonitrile. After sonication in an ice-water bath for 5 min, the mixture was vortexed and kept at −40 °C for 30 min. The samples were then centrifuged at 13,800 × *g* for 15 min at 4 °C. The supernatant was dried under a nitrogen stream and reconstituted in 80 μL of extraction solvent (methanol/acetonitrile/water, 2:2:1, v/v/v) containing isotope-labeled internal standards. After filtration through a 0.22 μm membrane, the samples were subjected to UHPLC–MS/MS analysis.

Preparation of XDD decoction samples: For XDD decoction sample preparation, 300 μL of XDD decoction was transferred into a microcentrifuge tube and centrifuged at 13,800 × *g* for 15 min at 4 °C. The supernatant was collected and mixed with 1,000 μL of extraction solvent (methanol/acetonitrile/water, 2:2:1, v/v/v) containing isotope-labeled internal standards. The mixture was sonicated in an ice bath for 5 min and incubated at −20 °C for 1 h to facilitate precipitation. After centrifugation under the same conditions, the final supernatant was collected for subsequent UHPLC–MS/MS analysis.

#### 2.5.3 UHPLC–MS/MS analysis

The chromatographic separation was carried out on a Vanquish UHPLC system using a Waters ACQUITY UPLC BEH Amide column (2.1 mm × 50 mm, 1.7 μm). Mobile phases contained two solvents: Solvent A which had 25 mmol/L of ammonium acetate as well as 25 mmol/L ammonia in water; and solvent B being Acetonitrile. A gradient elution program was executed according to Table 2. Autosampler temperature was kept at 4 °C, and injection volume was 2 μL.

**Table 2:** UHPLC gradient elution program.

No	Time	Flow (ml/min)	Event	%B
1	0.000		Run	
2	0.000	0.500		95.0
3	0.250	0.500		95.0
4	3.500	0.500		65.0
5	4.000	0.500		40.0
6	4.500	0.500		40.0
7	4.550	0.500		95.0
8	6.000	0.500		95.0
9	6.000		Stop Run	

Mass spectrometric analysis was carried out on an Orbitrap Exploris 120 mass spectrometer which was managed through the use of Xcalibur software (v4.4, Thermo Fisher Scientific). Full MS mode and data-dependent MS/MS mode were both adopted for detection. The following operation parameters were set: sheath gas flow rate at 50 Arb, auxiliary gas flow rate at 15 Arb, capillary temperature at 320 °C, full MS resolution at 60,000, and MS/MS resolution at 15,000. The normalized collision energy (SNCE)was stepped at 20/30/40, and the spray voltage was 3.8 kV in positive ion mode and −3.4 kV in negative ion mode.

#### 2.5.4 Network pharmacology analysis

Network pharmacology analysis was conducted by integrating data from various publicly accessible repositories. Targets related to XDD components were extracted from the Traditional Chinese Medicine Systems Pharmacology Database (TCMSP), ChEMBL, and STITCH. CIRI-related targets were obtained from GeneCards, Online Mendelian Inheritance in Man (OMIM), and the Therapeutic Target Database (TTD). Protein–protein interaction (PPI) data were acquired from STRING, and functional enrichment was performed using the Gene Ontology (GO) and Kyoto Encyclopedia of Genes and Genomes (KEGG) databases. Gene symbols were standardized according to UniProt.

Components of XDD identified in serum and/or tissues through UHPLC–MS/MS were evaluated for drug-likeness via the ADMET-AI platform. Candidates that met Lipinski’s rule of five, with oral bioavailability (OB) ≥ 30% and a quantitative estimate of drug-likeness (QED) ≥ 0.5, were selected for further study. Potential targets for these compounds were predicted and integrated using TCMSP, ChEMBL (confidence ≥ 80%), and STITCH (interaction score > 300). Disease-related targets were retrieved by searching “stroke” across multiple databases. After standardization through UniProt, the overlap between component- and disease-related targets was identified as potential therapeutic targets.

The intersecting targets were analyzed using the STRING database (*Homo sapiens* species only) to create a PPI network. An interaction confidence threshold of 0.7 was applied. Network topological parameters were calculated with the NetworkAnalyzer plugin in Cytoscape (v3.9.1), and core targets were chosen based on their Degree centrality. Functional enrichment for GO and KEGG pathways was performed on the potential therapeutic targets, with a *p* value < 0.05 considered statistically significant.

A multilayer network, illustrating the component–target–pathway interactions, was constructed to comprehensively depict the mechanisms of XDD against CIRI. This network was built upon the highest-ranked serum and/or tissue-absorbed components (by Degree), significantly enriched pathways, and their corresponding targets. Network visualizations were generated using Cytoscape (v3.9.1), with additional graphical outputs produced via the R packages ggplot2 (v3.5.0) and UpSetR (v1.4.0).

### 2.6 In vivo assessment of XDD efficacy in MCAO/R rats

#### 2.6.1 Animal grouping, model establishment, and treatment

Of the 94 rats entering the efficacy experiment, 15 were assigned to the sham-operated group, whereas the remaining 79 underwent MCAO/R surgery. After fasting for 12 h, rats were anesthetized with 1% sodium pentobarbital (40 mg/kg, i.p.). Except for rats in the sham-operated group, focal cerebral ischemia was induced using a modified intraluminal filament MCAO procedure. The left common carotid, external carotid, and internal carotid arteries were exposed, and a nylon filament was introduced through the external carotid artery to occlude the middle cerebral artery. The filament was maintained for 90 min and then withdrawn to initiate reperfusion. Rats in the sham-operated group underwent the same surgical exposure without middle cerebral artery occlusion. Body temperature was maintained at 37.0 °C–37.5 °C throughout surgery and recovery using a thermostatically controlled heating pad.

Following recovery from anesthesia and before the first treatment administration, neurological deficits were assessed using the Zea–Longa scoring system to evaluate successful model establishment. Rats with a Longa score of 1–3 were considered successfully modeled. Four rats were excluded because they did not meet the predefined model-success criterion (Longa score outside the range of 1–3). The remaining 75 successfully modeled rats were randomly allocated, using a random-number table, to the MCAO/R, edaravone, XDD low-dose (XDD-L), XDD medium-dose (XDD-M), and XDD high-dose (XDD-H) groups (*n* = 15 per group).

XDD was administered by oral gavage at doses of 0.95, 1.9, or 3.8 g/kg once daily for 7 consecutive days, with the first dose administered 1 h after the onset of reperfusion. Edaravone was administered intraperitoneally at 3.2 mg/kg once daily for 7 consecutive days, with the first dose also administered 1 h after reperfusion. Rats in the sham-operated and MCAO/R groups received an equivalent volume of distilled water by oral gavage according to the same treatment schedule. Neurological scoring, infarct-volume quantification, histological evaluation, and ELISA measurements were performed by investigators blinded to group allocation. Group codes were revealed only after completion of data collection and statistical analysis.

#### 2.6.2 Neurological deficit scoring

Neurological function was evaluated at 24 h after reperfusion as an efficacy outcome using the Zea–Longa scoring system (Longa et al., 1989). This assessment was distinct from the initial postoperative screening used to confirm successful MCAO/R model establishment, and the screening scores were not included in the efficacy analysis. Neurological deficits were scored on a 0–4 scale as follows: 0, no neurological deficit; 1, failure to fully extend the contralateral forelimb; 2, circling toward the paretic side; 3, falling toward the paretic side; and 4, inability to walk spontaneously or loss of consciousness.

#### 2.6.3 Sample collection and preparation

After anesthesia, blood was drawn and let stand at room temperature for 1 h before being centrifuged at 3,000 rpm for 15 min to get serum, which was stored at −80 °C. Brains that were designated for TTC staining were stored at −20 °C; brains used for H&E and Nissl staining were fixed in 4% paraformaldehyde. Cecal contents were collected and preserved at −80 °C until analyzed.

#### 2.6.4 Hemorheological analysis

Whole blood viscosity (low shear, moderate shear and high shear) and plasma viscosity were measured with an SA6600 automatic hemorheology analyzer. Whole blood viscosity at a shear rate of 200 s^−1^ was taken as the high-shear viscosity, while whole blood viscosity at a shear rate of 30 s^−1^ and 3 s^−1^ was taken as the medium-shear and low-shear viscosities, respectively.

#### 2.6.5 2,3,5-Triphenyltetrazolium chloride (TTC) staining

Rat brain was preserved at −20 °C for 20 min, then sliced into 5 coronal slices with a thickness of 2 mm. The slices were incubated in a 2% TTC solution at 37 °C for 30 min and flipped midway through to allow for even staining. Fixation was carried out using 4% paraformaldehyde for a duration of 24 h followed by image capturing and analysis via ImageJ software. Infarct volume (%) = ischemic volume of affected hemisphere/total brain volume × 100%* (Shang et al., 2025).

#### 2.6.6 H&E and Nissl staining

Whole brains and intestinal tissues were fixed with 4% paraformaldehyde, dehydrated with a series of ethanol, cleared in xylene, embedded in paraffin and sectioned. Intestinal tissues were stained by H&E; brain tissues were stained by both H&E and Nissl (toluidine blue). Morphological changes and neuronal injuries were observed using light microscope.

#### 2.6.7 Enzyme-linked immunosorbent assay (ELISA)

Serum levels of IL-1β and IL-6 were determined using ELISA kits in accordance with the manufacturer’s protocol.

#### 2.6.8 16S rDNA gene sequencing

Cecal samples were collected under sterile conditions and stored at −80 °C until analysis. Total microbial DNA was extracted, and DNA concentration and quality were assessed using a NanoDrop spectrophotometer and agarose gel electrophoresis. The V3–V4 hypervariable region of the bacterial 16S rRNA was amplified using the primers 338F and 806R. Purified amplicons were used for library construction and subjected to paired-end sequencing (2 × 250 bp) on an Illumina NovaSeq platform. The sequencing workflow included library quality assessment and paired-end sequencing using the NovaSeq PE250 strategy.

Raw sequencing reads were processed using a QIIME2-based bioinformatic pipeline. Primer sequences were removed using Cutadapt v4.9, followed by quality filtering, denoising, paired-end merging, and chimera removal using DADA2 v1.26.0. Amplicon sequence variants (ASVs) were generated after denoising, and singleton ASVs were removed before downstream analysis.

Taxonomic classification was performed in QIIME2 v2024.02 using the Greengenes database (Release 13.8), with a classification confidence threshold of 0.7. ASVs occurring in fewer than two samples were filtered out, and the ASV table was rarefied to the minimum sequencing depth among samples before diversity analyses.

Alpha diversity was evaluated using the Chao1, Shannon, and Simpson indices. Beta diversity was assessed based on Bray–Curtis, weighted UniFrac, unweighted UniFrac, and Jaccard distance matrices and visualized using principal coordinates analysis (PCoA) and non-metric multidimensional scaling (NMDS). Differences in microbial community structure among groups were further evaluated using analysis of similarities (ANOSIM) and permutational multivariate analysis of variance (PERMANOVA/Adonis).

Differentially abundant bacterial taxa were identified using linear discriminant analysis effect size (LEfSe), with an LDA score >3.0 and *p* < 0.05 considered significant.

Thirty cecal samples were initially submitted for 16S rRNA gene sequencing, including six biological samples from each of the Sham, MCAO/R, XDD-L, XDD-M, and XDD-H groups. The edaravone group was not included in the microbiome analysis. Seven samples affected by abnormal freeze–thaw handling were excluded before downstream analysis. Consequently, 23 samples were retained for the final microbiome analysis (Sham, *n* = 6; MCAO/R, *n* = 3; XDD-L, *n* = 4; XDD-M, *n* = 6; and XDD-H, *n* = 4).

#### 2.6.9 Untargeted metabolomics analysis

Approximately 25 ± 1 mg of each cecal sample was mixed with 500 μL of extraction solvent containing isotope-labeled internal standards (methanol/acetonitrile/water, 2:2:1, v/v/v). Samples were vortexed for 30 s, homogenized at 35 Hz for 4 min, and sonicated in an ice-water bath for 5 min; the homogenization–sonication procedure was repeated three times. After incubation at −40 °C for 1 h, 200 μL of the extract was filtered through a 0.22-μm filter plate under positive pressure (6 psi for 180 s), and the filtrate was collected for LC–MS analysis. Pooled quality-control (QC) samples were prepared by mixing equal aliquots from all experimental samples.

LC–MS analysis was performed using a Vanquish UHPLC system coupled to an Orbitrap Exploris 120 mass spectrometer (Thermo Fisher Scientific). Chromatographic separation was achieved using a Waters ACQUITY UPLC BEH Amide column (2.1 × 50 mm, 1.7 μm). Mobile phase A consisted of water containing 25 mmol/L ammonium acetate and 25 mmol/L ammonium hydroxide, whereas mobile phase B was acetonitrile. The autosampler was maintained at 4 °C, and the injection volume was 2 μL. Mass-spectrometric data were acquired under both positive- and negative-ion modes using Xcalibur v4.4. The sheath gas and auxiliary gas flow rates were set to 50 and 15 Arb, respectively; the capillary temperature was 320 °C; the full-MS and MS/MS resolutions were 60,000 and 15,000, respectively; stepped normalized collision energies were set at 20, 30, and 40; and spray voltages were 3.8 kV and −3.4 kV in positive- and negative-ion modes, respectively.

Raw LC–MS data were converted to mzXML format using ProteoWizard v3.0.24054 and processed using an in-house R-based pipeline. Features with an RSD > 30% or a missing rate >50% were excluded. Missing values were imputed using one-half of the minimum observed value, followed by total ion current (TIC) normalization. Of the 67,727 initially detected features, 41,906 were retained after preprocessing.

Metabolites were annotated against BiotreeDB v3.0 based on retention time (RT), precursor-ion MS1 information, and MS/MS fragmentation spectra. Identification confidence was classified according to the Metabolomics Standards Initiative (MSI): Level 1 indicated matching of MS1, MS2, and RT to authentic standards; Level 2 indicated matching of MS1 and MS2 to public databases; Level 3 indicated matching of MS1, MS2, and predicted RT to theoretical databases; and Level 4 represented unknown features.

Multivariate analyses were performed using SIMCA v18.0.1. Principal component analysis (PCA) was conducted after logarithmic transformation and centering. Orthogonal projections to latent structures–discriminant analysis (OPLS-DA) was performed after logarithmic transformation and unit-variance scaling, and model robustness was evaluated by seven-fold cross-validation and 200 permutation tests. Differential metabolites were initially screened using a variable importance in projection (VIP) value >1 and a Student’s *t*-test *p* value <0.05. Pathway annotation and enrichment analyses were performed using the KEGG database. Six biological replicates per group were initially subjected to untargeted metabolomic profiling. Following exploratory data inspection, the final numbers of samples retained for metabolomic analysis were Sham, *n* = 6; MCAO/R, *n* = 5; XDD-L, *n* = 4; XDD-M, *n* = 5; and XDD-H, *n* = 3.

#### 2.6.10 Integrated microbiome–metabolome analysis

To investigate the associations between gut microbial alterations and metabolic changes, an integrative analysis of the 16S rRNA gene sequencing and untargeted metabolomics datasets was performed. The two datasets were matched according to the original animal/sample identifiers. Only samples for which valid microbiome and metabolomics data were both available from the same individual rat were included. A total of 20 paired biological samples were retained for the final integrative analysis (Sham, *n* = 6; MCAO/R, *n* = 3; XDD-L, *n* = 3; XDD-M, *n* = 5; and XDD-H, *n* = 3).

Differential metabolites were initially identified based on VIP > 1 and *p* < 0.05. Metabolites showing opposite directions of change between disease modeling and XDD treatment were considered to exhibit XDD-associated reversal trends. Among these metabolites, the magnitude of reversal was evaluated according to |log_2_FC|, and 14 metabolites showing the most pronounced reversal-associated changes were selected for integrative analysis. Differentially abundant bacterial genera identified by 16S rRNA gene sequencing were subsequently correlated with these metabolites. Spearman’s rank correlation coefficients were calculated using R v3.6.3, with |*r*| > 0.5 and *p* < 0.05 considered significant. The resulting associations were interpreted as exploratory statistical co-variation rather than evidence of direct biological interaction or causality.

### 2.7 Statistical analysis

Statistical analyses of conventional pharmacodynamic and biochemical outcomes were performed using GraphPad Prism v10.5.0 and SPSS. Data distributions and homogeneity of variance were assessed before inferential testing. For normally distributed data with homogeneous variances, comparisons among multiple groups were performed using one-way analysis of variance (ANOVA), followed by Tukey’s multiple-comparison test. For data that did not meet parametric assumptions, the Kruskal–Wallis test was used, followed by an appropriate *post hoc* multiple-comparison test. A two-sided *p* value <0.05 was considered statistically significant. Unless otherwise specified, each biological replicate represented an independent animal (see Figure 1).

**Figure 1:**
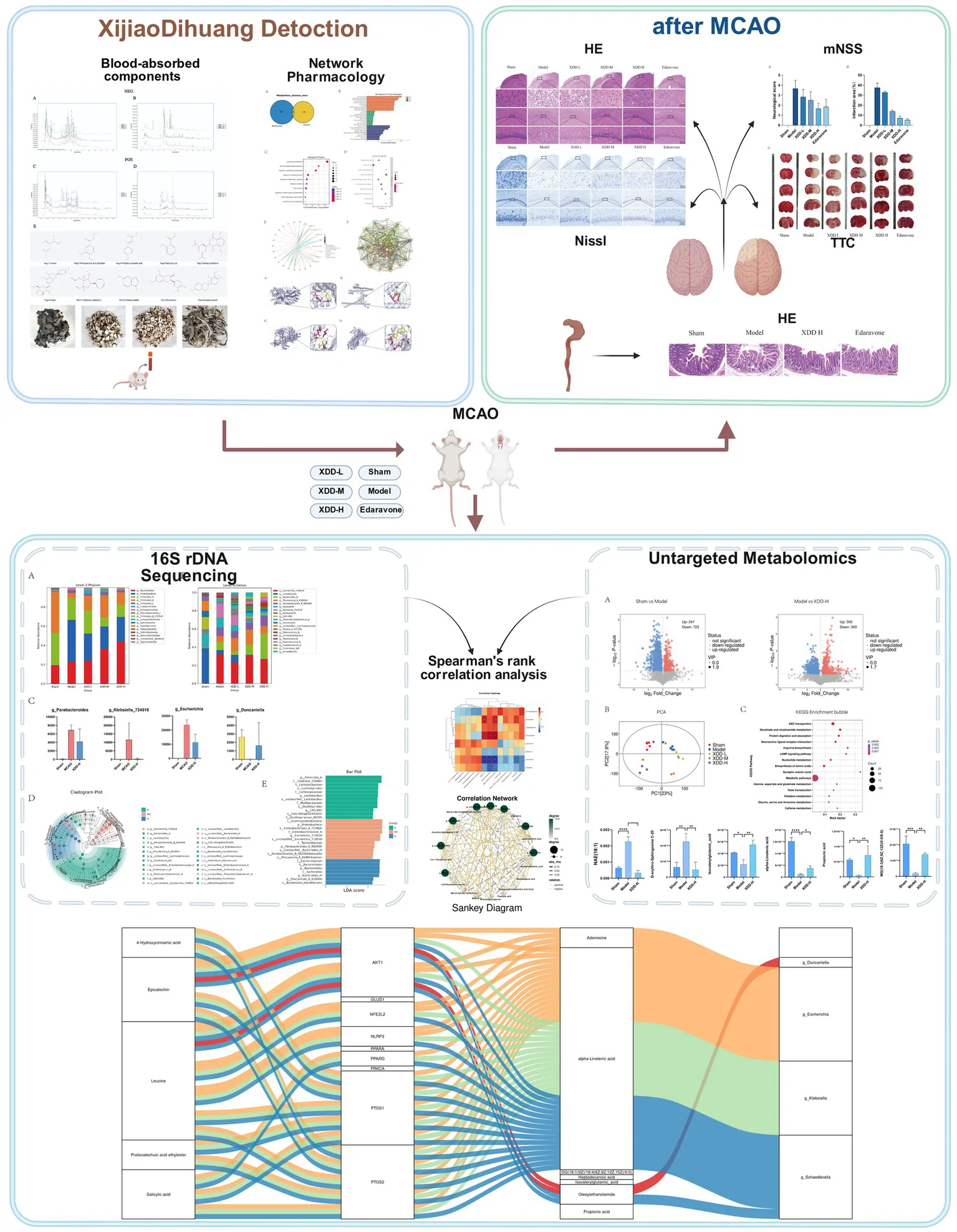
Study flowchart.

## 3 Results

### 3.1 Analysis of XDD-derived circulating components

Using UHPLC-QE-MS/MS, 54 XDD-derived constituents were consistently detected in medicated serum, while none were found in either blank serum or sham-treated control serum. Among these, the top 30 identified compounds were categorized into seven major chemical categories: sesquiterpenoids and phenylpropanoids (14 compounds), terpenoids (7 compounds), polyketides (3 compounds), alkaloids (2 compounds), and fatty acids (2 compounds), along with one amino acid–related molecule and one hybrid structural compound (Table 3).

**Table 3:** UHPLC–MS/MS-based identification of XDD blood components (Top 30).

No.	Recommended compound name	Formula	RT (s)	Measured m/z	Adduct	ppm error (absolute in source table)	MS/MS fragments	MS2 score	MSI level	Superclass
1	Acteoside	C29H36O15	279.1	623.198	[M−H]−	0.3	161;623.2;461.2	3.850	level1	Shikimates and Phenylpropanoids
2	Isoacteoside	C29H36O15	290.0	623.198	[M−H]−	0.0	161;623.2;461.2	3.940	level1	Shikimates and Phenylpropanoids
3	4-Hydroxybenzoic acid	C7H6O3	249.9	137.024	[M−H]−	0.1	93;137;94	3.940	level1	Shikimates and Phenylpropanoids
4	Loganic acid	C16H24O10	222.3	375.130	[M−H]−	0.1	169.1;213.1;375.1	3.910	level1	Terpenoids
5	Acetovanillone	C9H10O3	281.5	165.056	[M−H]−	0.2	150;165.1;151	3.960	level1	Shikimates and Phenylpropanoids
6	Vanillic acid	C8H8O4	257.5	167.035	[M−H]−	0.0	152;167;108	3.890	level1	Shikimates and Phenylpropanoids
7	Benzoylpaeoniflorin	C30H32O12	348.5	583.182	[M−H]−	0.3	121;165.1;582.3	3.970	level1	Terpenoids
8	Ferulate	C10H10O4	293.2	193.051	[M−H]−	0.3	134;178;193.1	3.920	level1	Shikimates and Phenylpropanoids
9	3,4-Dihydroxybenzoic acid (Protocatechuic acid)	C7H6O4	206.9	153.019	[M−H]−	0.2	153;109;108	3.990	level1	Shikimates and Phenylpropanoids
10	Paeoniflorin	C23H28O11	268.7	479.156	[M−H]−	0.6	121;165.1;327.1	3.990	level1	Terpenoids
11	Caffeic acid	C9H8O4	263.5	179.035	[M−H]−	0.3	135;179;136	3.980	level1	Shikimates and Phenylpropanoids
12	Catalpol	C15H22O10	52.5	361.114	[M−H]−	0.1	97;169.1;85	3.990	level1	Terpenoids
13	Paeonol	C9H10O3	210.1	167.070	[M + H]+	0.3	149.1;167.1;121.1	3.720	level1	Shikimates and Phenylpropanoids
14	2′,4’-Dihydroxyacetophenone	C8H8O3	299.7	151.040	[M−H]−	0.0	151;108;109	3.970	level1	Shikimates and Phenylpropanoids
15	Catechin	C15H14O6	241.6	291.086	[M + H]+	0.9	139;123;165.1	3.940	level1	Shikimates and Phenylpropanoids
16	Kaempferol	C15H10O6	369.2	285.041	[M−H]−	0.5	285;284.3;151	3.960	level1	Shikimates and Phenylpropanoids
17	Benzoyloxypaeoniflorin	C30H32O13	326.6	599.177	[M−H]−	0.1	137;599.2;121	3.920	level1	Terpenoids
18	Salidroside	C14H20O7	224.1	299.114	[M−H]−	0.5	59;89;71	3.860	level1	Shikimates and Phenylpropanoids
19	Taxifolin	C15H12O7	296.0	303.051	[M−H]−	0.2	303;125;285	3.860	level1	Shikimates and Phenylpropanoids
20	2,3-Dihydroxy-4-methoxyacetophenone	C9H10O4	234.0	183.065	[M + H]+	0.6	183.1;43;141.1	3.830	level1	Shikimates and Phenylpropanoids
21	Epicatechin	C15H14O6	257.6	291.086	[M + H]+	1.8	139;123;165.1	3.940	level1	Shikimates and Phenylpropanoids
22	(±)-Naringenin	C15H12O5	356.8	273.075	[M + H]+	1.4	273.1;153;147	3.950	level1	Shikimates and Phenylpropanoids
23	Rehmannioside D	C27H42O20	176.2	687.233	[M + H]+	1.2	165.1;147;85	3.920	level1	Terpenoids
24	galloylpaeoniflorin	C30H32O15	288.4	615.170	[M−H2O + H]+	1.7	153;179.1;151.1	3.940	level1	Terpenoids
25	Paeonolide	C20H28O12	249.8	459.151	[M−H]−	0.4	165.1;89;71	3.970	level1	Polyketides
26	Verbascoside	C29H36O15	290.0	625.212	[M + H]+	1.9	163;325.1;146.1	3.930	level1	Shikimates and Phenylpropanoids
27	Methylgallate	C8H8O5	243.7	183.030	[M−H]−	0.6	183;124;168	3.900	level1	Shikimates and Phenylpropanoids
28	5-Hydroxymethylfurfural	C6H6O3	98.4	127.039	[M + H]+	1.1	127;109;99	3.960	level1	Polyketides
29	3-Hydroxy-4-methoxyacetophenone	C9H10O3	253.5	167.070	[M + H]+	2.1	167.1;43;149.1	3.940	level1	Shikimates and Phenylpropanoids
30	Dihydroferulic acid	C10H12O4	280.3	195.066	[M−H]−	0.1	136.1;195.1;123	3.880	level1	Shikimates and Phenylpropanoids

The total ion chromatograms (TIC) from *in vitro* XDD samples and corresponding medicated serum samples showed a high degree of overlap (Figure 2). The mass-to-charge ratio (m/z) difference is controlled within 5 ppm, with the retention time variation for each analyte maintained within 0.3 min, demonstrating the reliability of our analytical method and ensuring accurate compound identification.

**Figure 2:**
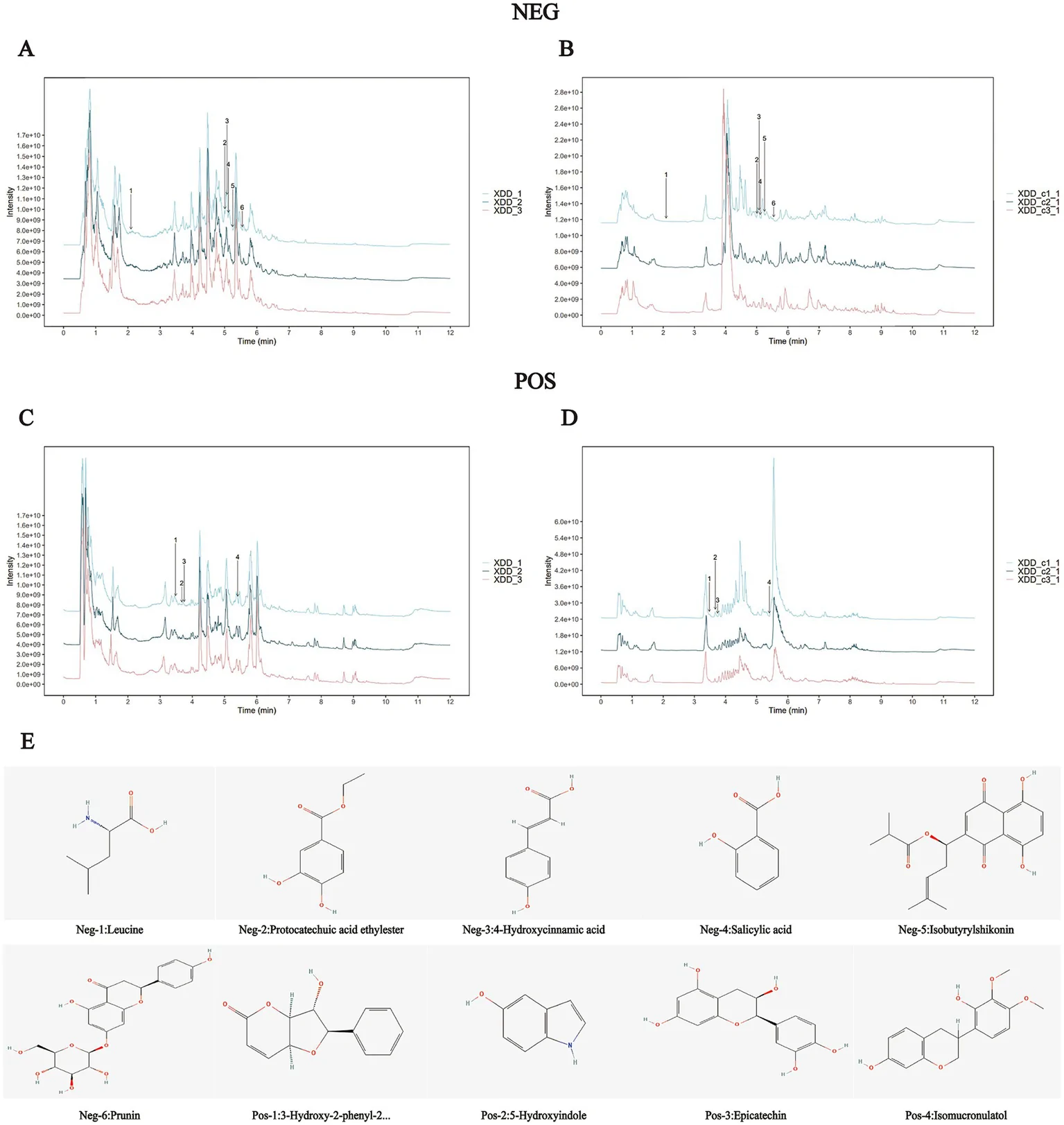
TIC peak plots of XDD decoction and XDD-containing serum samples under positive and negative ion modes. **(A)** Total ion current profile of the XDD decoction in positive ion mode; **(B)** Total ion current profile of the XDD decoction in negative ion mode; **(C)** Positive ion mode ion current profiles of serum samples containing the drug from rats in the XDD group; **(D)** Negative ion mode ion current profiles of serum samples containing the drug from rats in the XDD group; **(E)** TIC with annotated chemical structure (refer to the accompanying table for details).

### 3.2 Integrated network pharmacology and serum metabolomics analysis

A total of 783 putative targets associated with XDD-derived circulating components and 136 CIRI-related targets were identified. Intersection analysis revealed 20 shared targets between XDD and CIRI (Figure 3A). Based on these overlapping targets, Gene Ontology (GO) enrichment analysis identified the top 10 significantly enriched biological processes (*p* < 0.05), including epithelial cell proliferation, extrinsic apoptotic signaling pathway, regulation of epithelial cell proliferation, response to xenobiotic stimulus, regulation of inflammatory response, and response to hypoxia (Figure 3B). These enriched terms were mainly related to inflammation, apoptotic regulation, hypoxia response, and cell proliferation. Kyoto Encyclopedia of Genes and Genomes (KEGG) pathway enrichment analysis further showed that the intersecting targets were significantly enriched in pathways such as proteoglycans in cancer, pathways in cancer, longevity regulating pathway, lipid and atherosclerosis, and fluid shear stress and atherosclerosis (Figure 3C). In addition, the protein–protein interaction (PPI) network of the overlapping targets is shown in Figure 3D.

**Figure 3:**
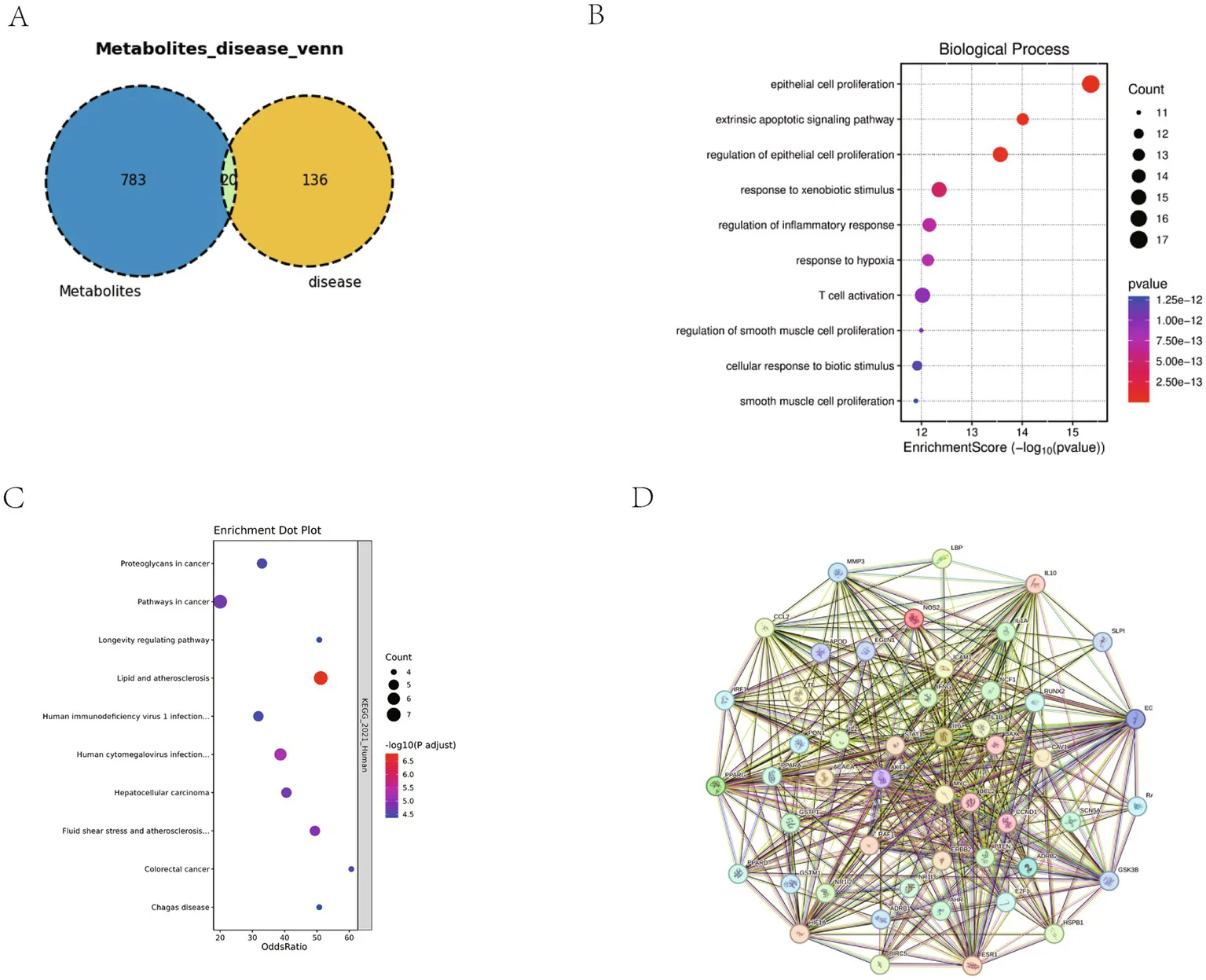
Network pharmacology analysis of the common targets of XDD and CIRI. **(A)** Venn diagram of putative targets of XDD-derived circulating components and CIRI-related targets; **(B)** GO enrichment analysis of the overlapping targets; **(C)** KEGG pathway enrichment analysis of the overlapping targets; **(D)** PPI network of the overlapping targets.

Protein–protein interaction (PPI) network analysis showed that the top 10 nodes ranked by degree included EGR1, AKT1, NLRP3, PTGS1, HMOX1, and CCL2, among others (Figure 3D). AKT1 and NLRP3 exhibited particularly high degree values, suggesting that they may function as central regulatory nodes potentially mediating the therapeutic effects of XDD, though further investigation is required to confirm their roles.

To further clarify the rationale for molecular docking, representative XDD-derived compounds were reselected from the high-confidence circulating components after revision of Table 3. The selection was based on MSI identification level, mass accuracy within ± 5 ppm, MS/MS evidence, reproducible detection in XDD-containing serum, formula relevance, and exclusion of common endogenous metabolites with limited formula specificity. Among the retained candidates, epicatechin and protocatechuic acid were selected for docking because they were high-confidence phenolic compounds detected in XDD-containing serum, showed clear chemical relevance to XDD, and were connected to key targets in the component–target network. Therefore, epicatechin and protocatechuic acid were selected as representative bioactive compounds for molecular docking rather than all detected circulating constituents. HMOX1, PTGS1, and AKT1 were selected as representative targets because they were associated with oxidative stress response, inflammatory lipid mediator signaling, and cell survival/inflammatory regulation, respectively.

As shown in Figure 4, all selected ligand–target pairs yielded negative binding energies lower than −5.0 kcal/mol, suggesting favorable binding tendencies between the representative XDD-derived compounds and the predicted targets. Epicatechin showed strong docking affinities with AKT1, PTGS1, and HMOX1, with binding energies of −9.131, −8.395, and −7.831 kcal/mol, respectively. Protocatechuic acid also showed acceptable affinities with PTGS1, AKT1, and HMOX1, with binding energies of −6.350, −5.847, and −5.797 kcal/mol, respectively. These results suggest that epicatechin and protocatechuic acid may interact with targets involved in inflammatory regulation, lipid mediator metabolism, oxidative stress response, and cell-survival signaling. However, these docking results should be interpreted as computational evidence and require further experimental validation.

**Figure 4:**
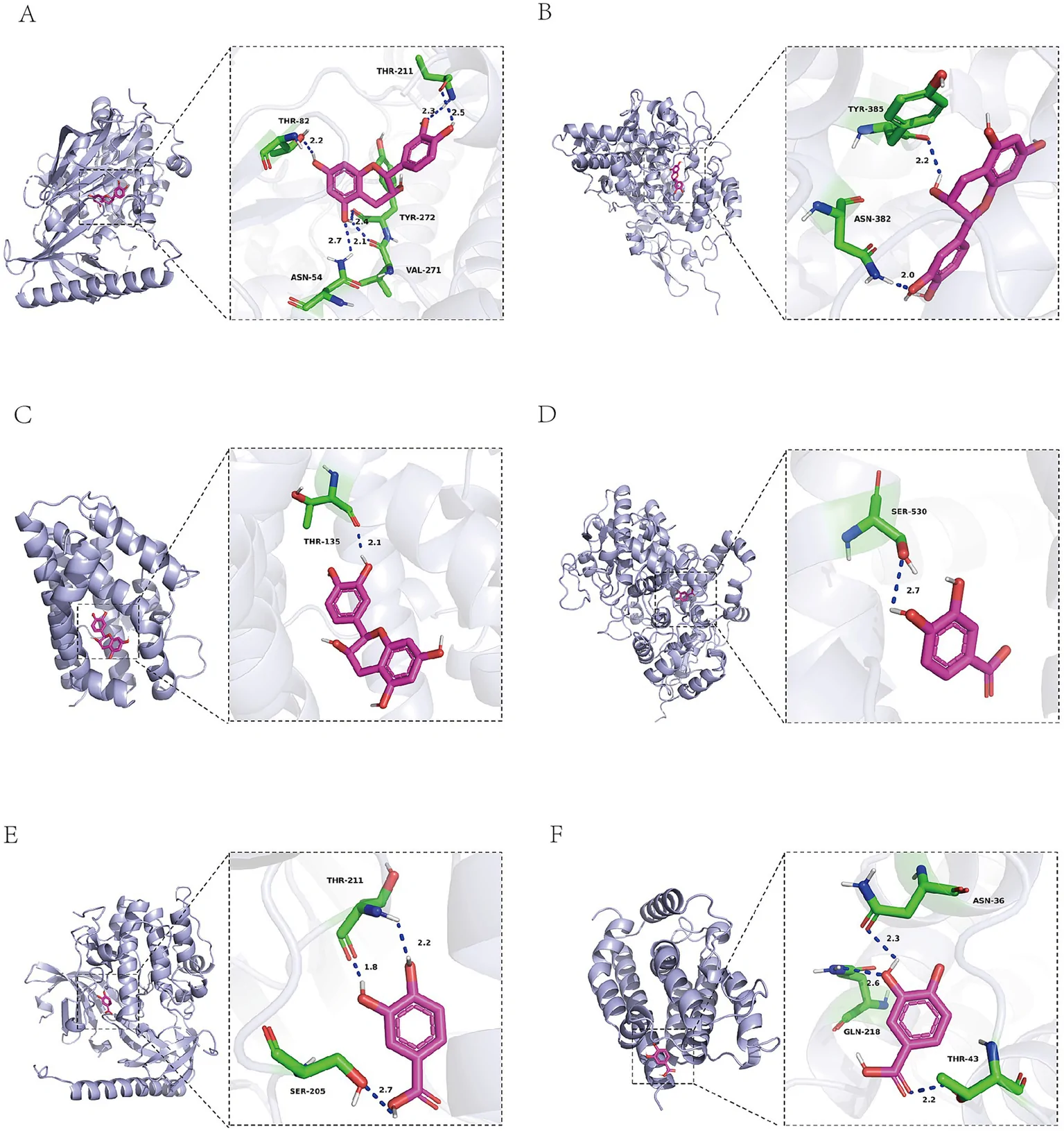
Molecular docking patterns of representative XDD-derived compounds with key target proteins. **(A)** AKT1–epicatechin (−9.131 kcal/mol); **(B)** PTGS1–epicatechin (−8.395 kcal/mol); **(C)** HMOX1–epicatechin (−7.831 kcal/mol); **(D)** PTGS1–protocatechuic acid (−6.350 kcal/mol); **(E)** AKT1–protocatechuic acid (−5.847 kcal/mol); **(F)** HMOX1–protocatechuic acid (−5.797 kcal/mol).

### 3.3 XDD improves neurological function and attenuates cerebral infarction

Neurological function was assessed 24 h after reperfusion. Neurological deficit scores differed significantly among the six experimental groups (Kruskal–Wallis H = 26.35, n = 15 per group, *p* < 0.0001; ε^2^ = 0.712). Dunn’s multiple-comparison test showed that neurological deficit scores were significantly higher in the MCAO/R group than in the sham group (mean rank difference = 27.42; Dunn-adjusted *p* < 0.0001). Compared with the MCAO/R group, the XDD-H group showed a significant reduction in neurological deficit scores (mean rank difference = 16.75; adjusted *p* = 0.0220), whereas the differences observed in the XDD-L (mean rank difference = 5.92; adjusted *p* > 0.9999), XDD-M (mean rank difference = 9.42; adjusted *p* = 0.5471), and edaravone groups (mean rank difference = 15.00; adjusted *p* = 0.0539) did not reach statistical significance (Figure 5A).

**Figure 5:**
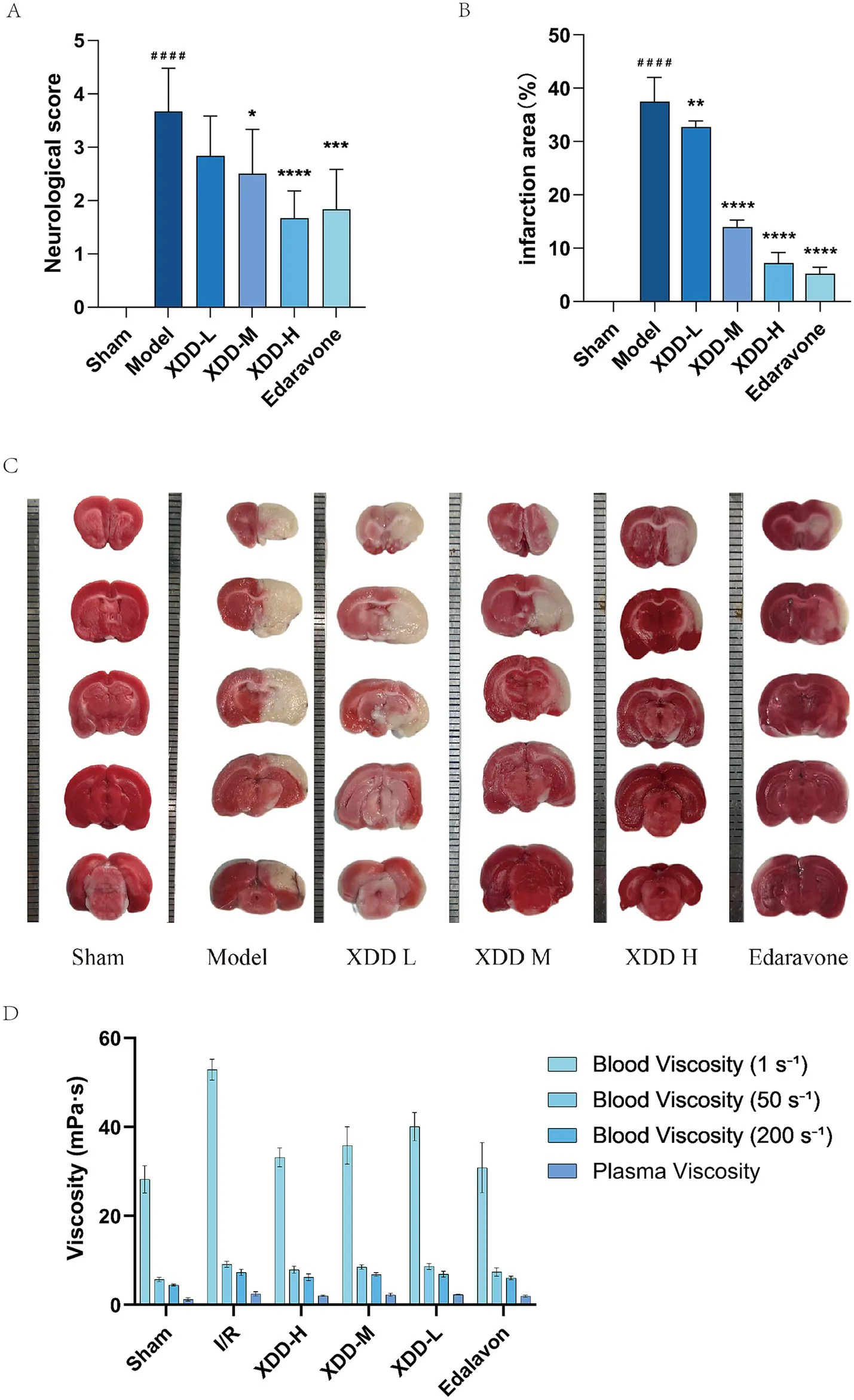
Impact of XDD on neurological function, brain tissue injury and blood flow characteristics in rats with CIRI. **(A)** The neurological deficit score recorded 24 h after MCAO/R (*n* = 15); **(B)** Quantification of infarcted brain tissue volume (*n* = 6); **(C)** Representative brain sections stained with TTC; **(D)** Plasma viscosity and whole blood viscosity at varying shear rates (low, medium, and high).

Consistent with the neurological findings, TTC staining showed no detectable infarction in the sham group, whereas marked cerebral infarction was observed in the MCAO/R group. Cerebral infarct volume differed significantly among groups. Relative to the MCAO/R group, infarct volume was lower in the XDD-L group by 4.78 percentage points (95% CI, 1.44–8.12; Dunnett-adjusted *p* = 0.0029), in the XDD-M group by 23.56 percentage points (95% CI, 20.22–26.90; adjusted *p* < 0.0001), and in the XDD-H group by 30.27 percentage points (95% CI, 26.93–33.61; adjusted *p* < 0.0001). Edaravone treatment was associated with a 32.32-percentage-point reduction relative to the MCAO/R group (95% CI, 28.98–35.66; adjusted *p* < 0.0001). The difference between the MCAO/R and sham groups was 37.51 percentage points (95% CI, 34.17–40.85; adjusted *p* < 0.0001) (Figures 5B, C; *n* = 6 per group).

Hemorheological analysis showed significantly increased whole-blood viscosity across the examined shear rates and increased plasma viscosity in the MCAO/R group compared with the sham group (*p* < 0.01). XDD treatment reduced these abnormalities, with the largest changes observed in the high-dose group (Figure 5D). These findings indicate that XDD attenuated MCAO/R-associated hemorheological disturbances.

### 3.4 XDD attenuates histopathological tissue injury

H&E staining of brain sections revealed that neurons within the hippocampus and cortex of the sham group were densely arranged, exhibiting intact morphology, uniform cytoplasm, and clearly defined nuclei and nucleoli, with no evidence of degeneration or necrosis. Conversely, the MCAO/R group displayed significant neuronal loss and disorganization, prominent vacuolation, and severe structural disruption, accompanied by nuclear pyknosis and necrotic features. Relative to the MCAO/R group, XDD-treated groups exhibited substantial histological improvement in the hippocampus, characterized by reduced vacuole formation and nuclear pyknosis, along with a more organized cellular arrangement. The cortical architecture was also better preserved, evidenced by decreased intercellular spaces, more uniform cytoplasm, clearer cell boundaries, and enhanced nuclear integrity. In subjects receiving high-dose XDD treatment and those in the edaravone group, the benefits became more obvious (Figure 6A).

**Figure 6:**
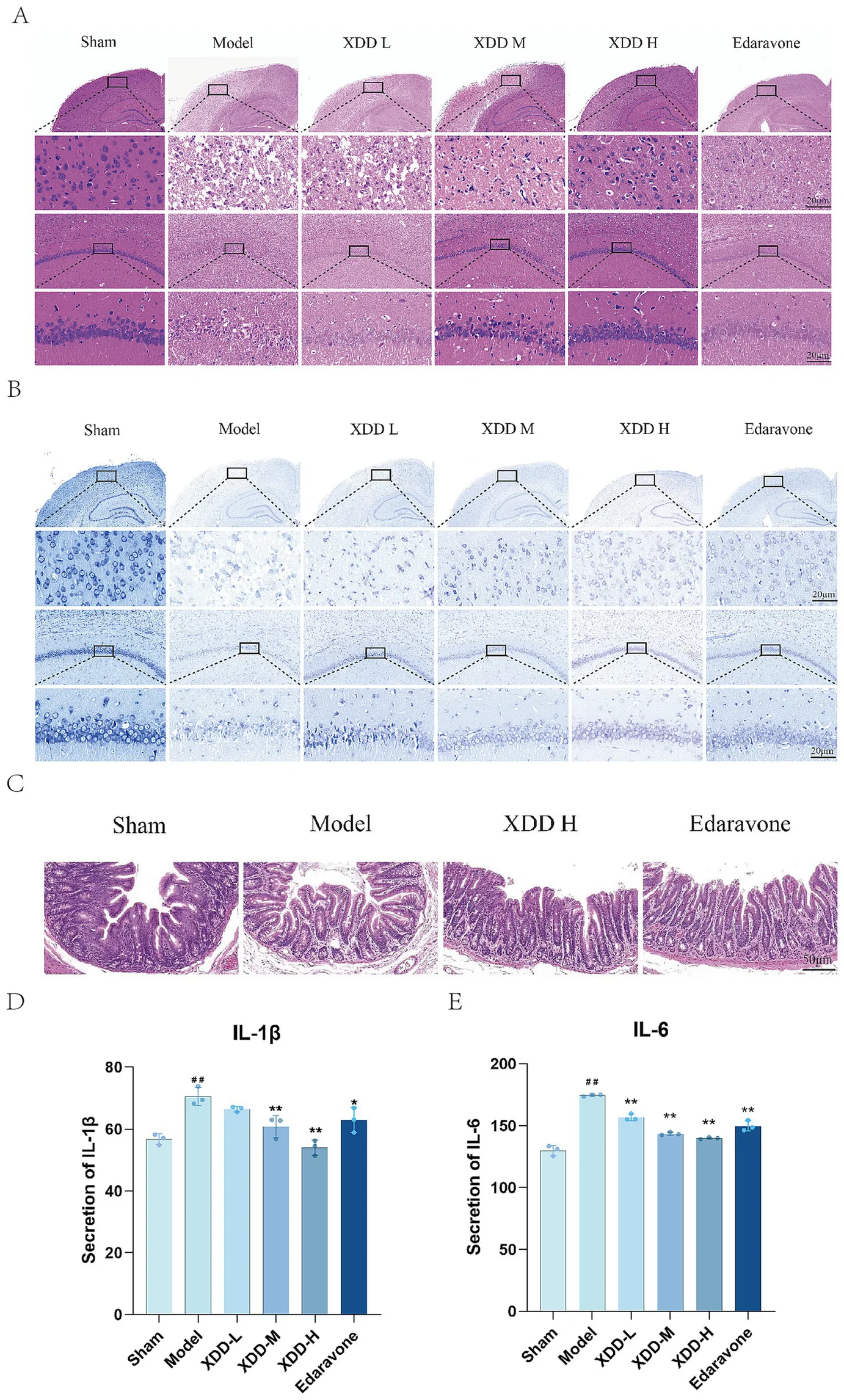
XDD attenuates brain and intestinal histopathology and reduces serum inflammatory mediators in rats with CIRI. **(A)** H&E staining of the cortex and hippocampus (scale bar = 20 μm); **(B)** Nissl staining of the cortex and hippocampus (scale bar = 20 μm); **(C)** H&E staining of the small intestine (scale bar = 50 μm); **(D)** Serum IL-1β levels; **(E)** Serum IL-6 levels.

Consistently, Nissl staining revealed large, regularly shaped neurons with densely distributed Nissl bodies in the sham group. In the MCAO/R group, Nissl bodies were sparse and irregular, with weak staining, a reduced Nissl-positive area, and prominent vacuolar degeneration. All treatment groups demonstrated varying degrees of morphological recovery in both hippocampal and cortical regions, including increased neuronal volume, enhanced staining intensity, higher Nissl body density, and more preserved cellular structures. The XDD-H and edaravone groups showed the most pronounced morphological preservation.(Figure 6B).

Furthermore, H&E staining of intestinal tissue demonstrated intact epithelial organization and preserved villus microvilli in the sham group. In comparison to the sham group, the MCAO/R group displayed broken villi in the small intestine, disordered and sparse glandular structures, reduced goblet cells, muscle layer damage, and the presence of cellular necrosis. Treatment with high-dose XDD or edaravone markedly improved intestinal architecture, resulting in partial restoration of microvilli and glandular organization (Figure 6C).

### 3.5 XDD reduces circulating inflammatory mediators

Serum inflammatory cytokine levels were assessed in 15 rats per group. IL-6 levels were significantly higher in the MCAO/R group than in the sham group, with a mean difference of 44.59 (95% CI, 38.12–51.06; Dunnett-adjusted *p* < 0.0001). Relative to the MCAO/R group, IL-6 levels were significantly lower in the XDD-L group (mean difference = 17.89, 95% CI, 11.42–24.36; adjusted *p* < 0.0001), XDD-M group (mean difference = 31.10, 95% CI, 24.63–37.57; adjusted *p* < 0.0001), XDD-H group (mean difference = 34.67, 95% CI, 28.20–41.14; adjusted *p* < 0.0001), and edaravone group (mean difference = 24.78, 95% CI, 18.31–31.25; adjusted *p* < 0.0001) (Figure 6E).

Serum IL-1β levels were also significantly elevated in the MCAO/R group compared with the sham group (mean difference = 13.80, 95% CI, 7.15–20.44; Dunnett-adjusted *p* = 0.0003). Compared with the MCAO/R group, IL-1β levels were significantly lower in the XDD-M group (mean difference = 9.69, 95% CI, 3.05–16.34; adjusted *p* = 0.0048), XDD-H group (mean difference = 16.50, 95% CI, 9.85–23.14; adjusted *p* < 0.0001), and edaravone group (mean difference = 7.59, 95% CI, 0.95–14.24; adjusted *p* = 0.0241). The difference between the XDD-L and MCAO/R groups was not statistically significant (mean difference = 4.03, 95% CI, −2.62 to 10.67; adjusted *p* = 0.3234) (Figure 6D).

### 3.6 XDD restores gut microbiota dysbiosis

To determine if XDD’s therapeutic impact on CIRI is linked to alterations in gut microbiota, we sequenced the cecal contents from the Sham, MCAO/R, and XDD treatment groups. Rarefaction analysis based on the Shannon index indicated that both the sequencing depth and sample size were adequate for subsequent analyses (Figure 7A).

**Figure 7:**
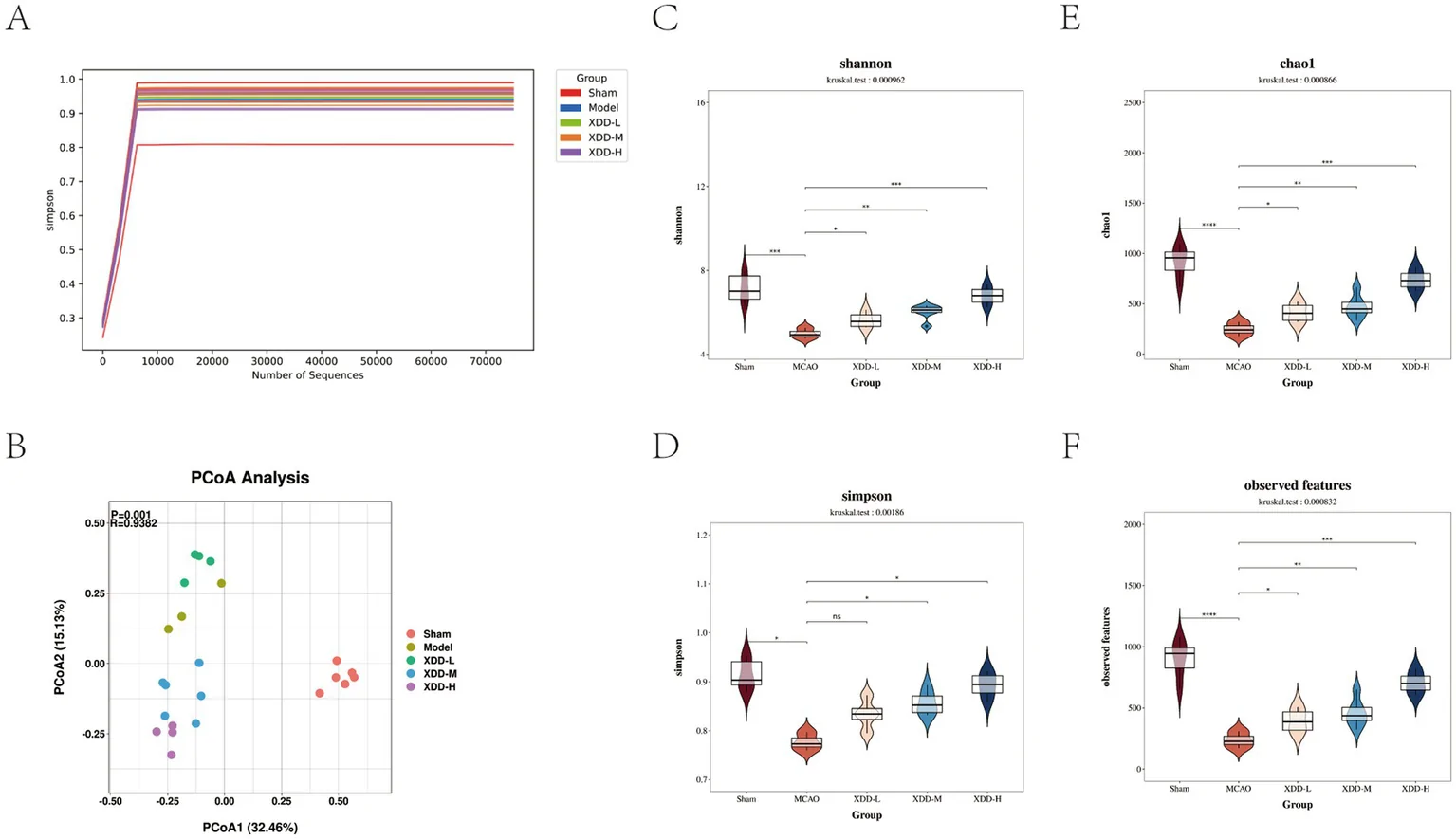
Effect of XDD on the diversity of intestinal microflora alpha and beta (Sham, *n* = 6; MCAO/R, *n* = 3; XDD-L, *n* = 4; XDD-M, *n* = 6; XDD-H, *n* = 4). **(A)** Sparse curve based on Shannon index; **(B)** PCoA; for beta diversity analysis; **(C)** Chao1 index; **(D)** Shannon diversity index; **(E)** Simpson diversity index; **(F)** Number of observed features.

Alpha diversity indices—including richness-related metrics (e.g., Chao1) and diversity/evenness measures (Shannon and Simpson)—revealed significant group-level differences (Figures 7C–E). Compared to the sham group, the MCAO/R group demonstrated significantly reduced richness and diversity (*p* < 0.05), suggesting that cerebral ischemia–reperfusion significantly reduced gut microbial diversity. After the intervention with XDD, alpha diversity showed improvement, especially at elevated doses, hinting at a return to a more diverse microbial environment.

Principal coordinated analysis (PCoA) and Non-metric Multidimensional Scaling (NMDS) show different clustering patterns. Sham group and MCAO/R group are obviously separated, while the samples processed by XDD are located in the middle (Figure 7B). Importantly, the middle-dose and high-dose XDD groups are closer to the sham group than the low-dose group, and further away from the MCAO/R group, which shows that XDD can gradually restore the structure of the whole microbial community according to the dose.

In the MCAO/R group, there was a lower relative abundance of Firmicutes and a higher presence of Proteobacteria and Patescibacteria at the phylum level compared to the sham group. These shifts were partially reversed by XDD administration, with the high-dose group demonstrating the most notable changes (Figure 8A). The MCAO/R group exhibited increased levels of *Escherichia*, *Parabacteroides*, and *Klebsiella*, while *Duncaniella*, *Lactobacillus*, and *Limosilactobacillus* were less prevalent than in the sham group (Figure 8B). XDD treatment mitigated some of these alterations. Notably, the differences in *Klebsiella* and *Duncaniella* between groups were particularly striking: *Klebsiella* was nearly absent in the Sham, XDD-M, and XDD-H groups but was abundant in the MCAO/R group, while *Duncaniella* exhibited the reverse pattern. These results indicated that XDD reduced the microbial imbalance in CIRI at phylum and genus taxonomic levels.

**Figure 8:**
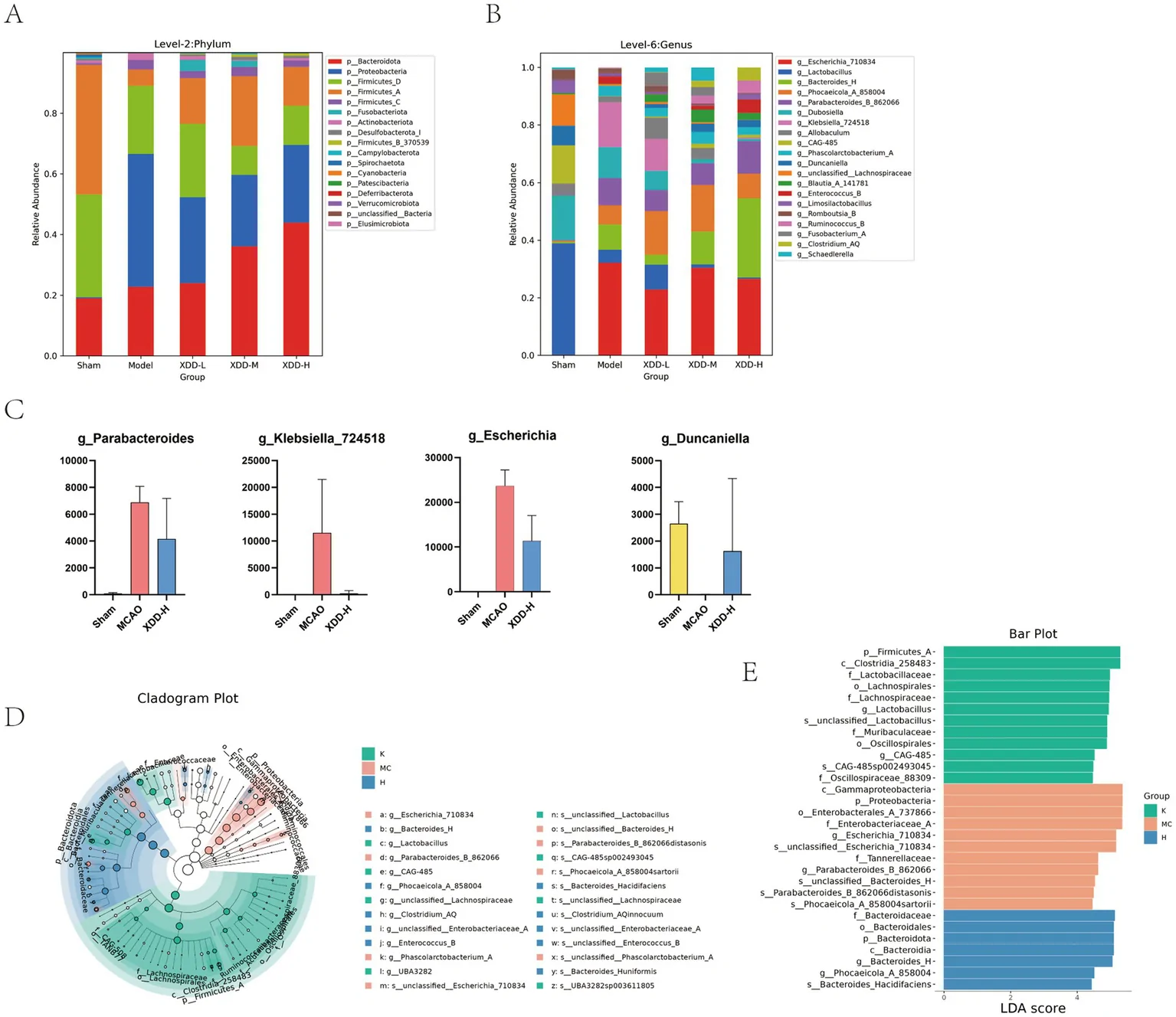
Gut microbiota composition and differential taxa among groups. **(A)** Relative abundance of gut microbial communities at the phylum level across experimental groups. **(B)** Relative abundance of gut microbial communities at the genus level across experimental groups. **(C)** Relative abundance of representative differentially abundant bacterial genera among groups. **(D)** LEfSe cladogram showing the phylogenetic distribution of differential microbial taxa. **(E)** LDA score distribution of significantly differential taxa identified by LEfSe analysis.

### 3.7 Differential metabolite profiling across groups

Untargeted LC-MS-based metabolomics revealed significant metabolic alterations across the experimental groups. In comparing the sham and MCAO/R groups, 247 metabolites were found to be upregulated, while 723 were downregulated. Similarly, in the comparison between the model and high-dose XDD groups, 300 metabolites were upregulated, and 390 were downregulated (Figure 9A). These results indicate that MCAO/R induced substantial metabolic reprogramming, with high-dose XDD partially reversing these changes.

**Figure 9:**
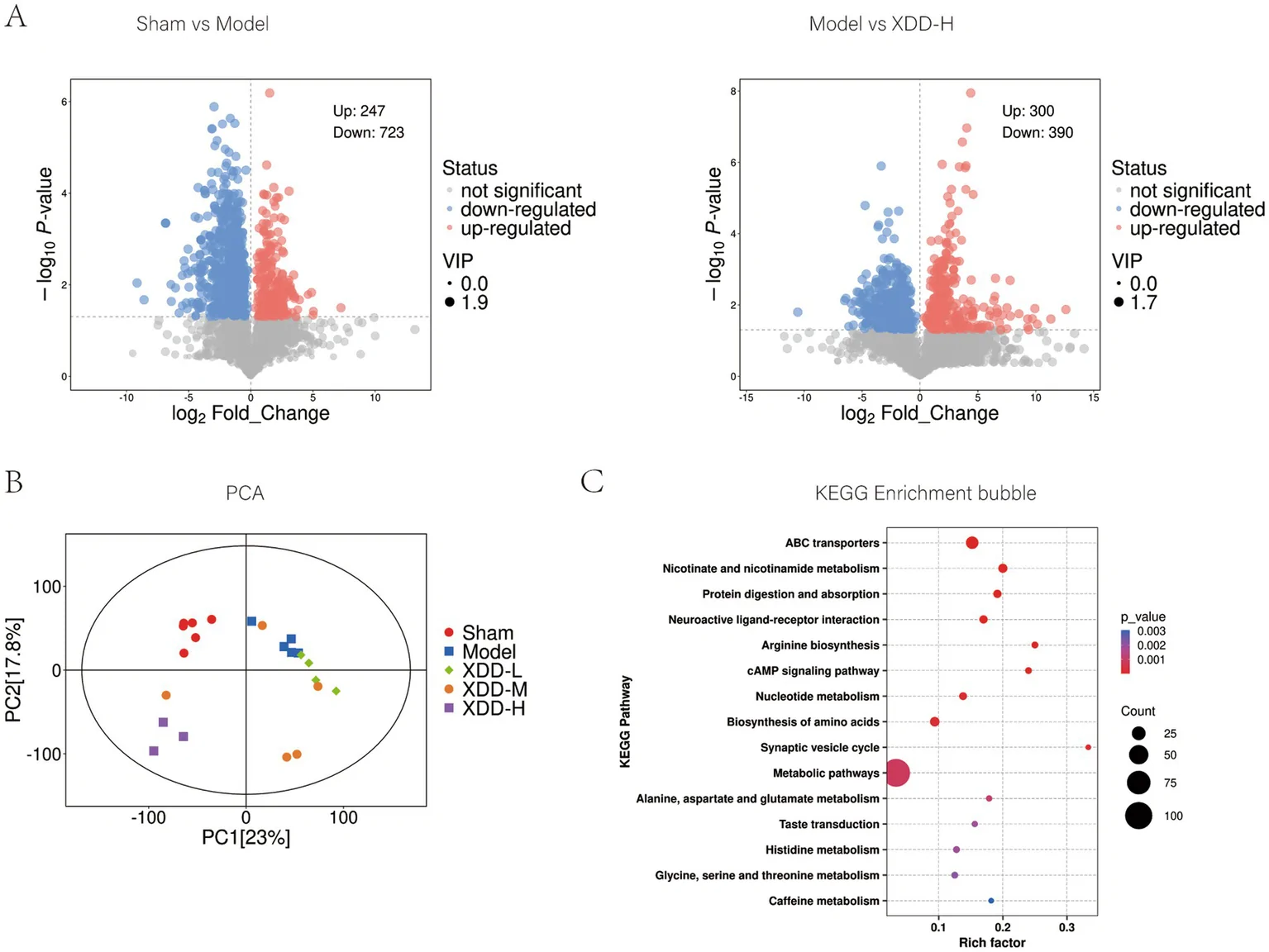
Differential metabolomic profiling among groups. **(A)** Volcano plots showing differential metabolites between the Sham and MCAO/R groups and between the MCAO/R and XDD-H groups. **(B)** Principal component analysis (PCA) score plot of cecal metabolomic profiles across experimental groups. **(C)** KEGG pathway enrichment analysis of differential metabolites.

Further pairwise comparisons among the XDD-H, XDD-M, and XDD-L groups demonstrated dose-dependent divergence in metabolic profiles, with 347 metabolites exhibiting bidirectional regulation following XDD treatment. Specifically, 143 metabolites increased after modeling but decreased after XDD administration, while 204 metabolites showed the opposite trend. This suggests a systematic normalization of metabolic processes following treatment.

To further evaluate the overall metabolic differences among groups, PCA was performed on the cecal metabolomic data. As shown in Figure 9B, the Sham and MCAO/R groups exhibited distinct separation, indicating that MCAO/R induced marked alterations in the global metabolic profile.

Significant enrichment in multiple pathways, mainly associated with metabolism, neurotransmission, and immune response, was revealed by KEGG enrichment analysis of differential metabolites from cecal samples. These included pathways related to ABC transporters, nicotinate and nicotinamide metabolism, protein digestion and absorption, and neuroactive ligand–receptor interactions (Figure 9C). Furthermore, important pathways related to amino acid metabolism, including arginine biosynthesis and the metabolism of alanine, aspartate, glutamate, and glycine/serine/threonine, were enriched as well. Notably, metabolites exhibiting reversal trends were associated with lipid metabolism pathways, including fatty acid degradation, glyceride metabolism, and sphingolipid metabolism (Table 4).

**Table 4:** Untargeted metabolomics analysis highlighted important metabolites.

Name	Formula	m/z	Rt	VIP	*p*	Fold change	Metabolic pathway
Heptadecanoic acid	C17H34O2	269.2486	20.6	1.844814114	7.49124E-05	3.599182536	Fatty acid degradation
NAE(18:1)	C20H39NO2	326.3053	18	1.844669751	1.29271E-06	0.128627083	Fatty acid metabolism
MG(18:3,62,92,122/0.00/0.0)	C21H36O4	353.2685	17.4	1.803258523	0.000317184	0.089196899	Metabolic pathways
Salpha-Linolenic acid	C18H30O2	277.2172	20.4	1.797827145	8.71647E-05	0.075695103	α-linolenic acid metabolism
1,24-Dihydroxyvitamin D3	C27H44O3	417.3364	18	1.713416509	0.00545609	1.678885808	Vitamin digestion and absorption
Hexadecatrienoylcarnitine (Cat(16:3))	C23H39NO4	394.2949	36.4	1.646994827	0.029837152	5.681218103	Fatty acid degradation
cis-11-Eicosenoic acid	C20H38O2	309.28	20.3	1.355031526	0.004129086	1.571427332	Fatty acid degradation
DG(18:1/92)/18:4 (62,92,122,152/0.0)	C39H66O5	615.4977	16.3	1.803258523	0.043180535	0.144212833	Glyceride metabolism
D-erythro-Sphingosine C-20	C20H41NO2	328.3208	30.1	1.481744943	0.001993889	3.022517806	Sphingolipid metabolism
N-Acetylglycylglycine	C6H10N2O4	175.0712	73.4	1.628867005	0.000219098	0.387742859	Glycine metabolism
Adenosine	C10H13N5O4	268.1039	76.2	1.490410673	0.018251494	0.237406113	Purine metabolism
Propionic acid	C3H6O2	73.0295	79.9	1.365552984	0.013201071	0.399528853	Short-chain fatty acid metabolism
Isovalerylglutamic acid	C10H17NO5	232.1179	70.3	1.362006379	0.008830706	0.709446935	Amino acid metabolism

### 3.8 Integrated 16S microbiome and Cecal metabolomics analysis

Spearman correlation analysis was performed between eight differentially abundant bacterial genera and 14 representative differential metabolites showing pronounced XDD-associated reversal trends. Significant associations between microbiota and metabolites were revealed by the analysis (Figure 10). Specifically, *Duncaniella*, *Limosilactobacillus*, *Schaedlerella*, and *Ligilactobacillus* exhibited positive correlations with MG(18:3(6Z,9Z,12Z)/0:0/0:0), α-linolenic acid, and γ-linolenic acid. This indicates that these taxa could be involved in the synthesis and/or metabolism of these lipids, and conversely, that these metabolites may influence bacterial growth or activity. Conversely, *Klebsiella*, *Blautia_A*, and *Escherichia* displayed significant negative correlations with these metabolites.

**Figure 10:**
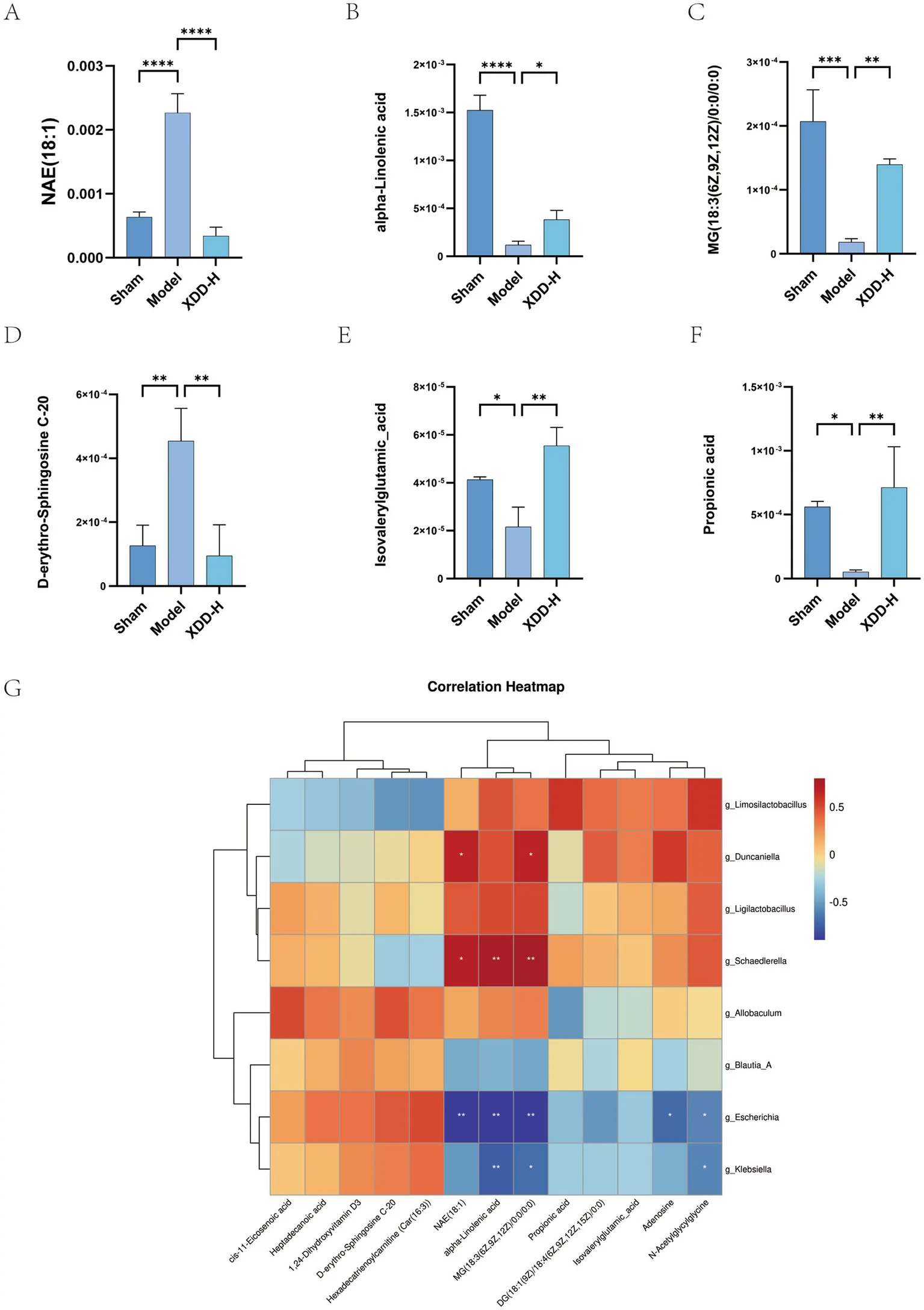
Integrated microbiome–metabolome analysis. Only paired samples with both valid 16S rRNA gene sequencing and metabolomics data from the same animals were included in the correlation analysis (total *n* = 20; Sham, *n* = 6; MCAO/R, *n* = 3; XDD-L, *n* = 3; XDD-M, *n* = 5; XDD-H, *n* = 3). **(A)** NAE(18:1). **(B)** α-Linolenic acid. **(C)** MG(18:3(6Z,9Z,12Z)/0:0/0:0). **(D)** D-erythro-sphingosine C-20. **(E)** Isovalerylglutamic acid. **(F)** Propionic acid. **(G)** Spearman correlation heatmap between eight differentially abundant bacterial genera and 13 representative differential metabolites.

### 3.9 Integrated microbiome–metabolome analysis

Using the 20 paired samples for which both valid 16S rRNA gene sequencing and metabolomics data were available (Sham, *n* = 6; MCAO/R, *n* = 3; XDD-L, *n* = 3; XDD-M, *n* = 5; and XDD-H, *n* = 3), Spearman correlation analysis was performed between eight differentially abundant bacterial genera and 14 representative differential metabolites showing pronounced XDD-associated reversal patterns.

After correction for multiple testing using the Benjamini–Hochberg false discovery rate (FDR) procedure, significant microbiota–metabolite associations were identified and visualized as a correlation heatmap (Figure 10). Duncaniella, Limosilactobacillus, Schaedlerella, and Ligilactobacillus showed positive associations with several lipid-related metabolites, including MG(18:3(6Z,9Z,12Z)/0:0/0:0), α-linolenic acid, and γ-linolenic acid, whereas Klebsiella, Blautia_A, and Escherichia showed negative associations with these metabolites. These findings indicate coordinated statistical co-variation between specific bacterial genera and lipid-related metabolites; however, they do not establish direct metabolic interactions or causal relationships (see Figure 11).

**Figure 11:**
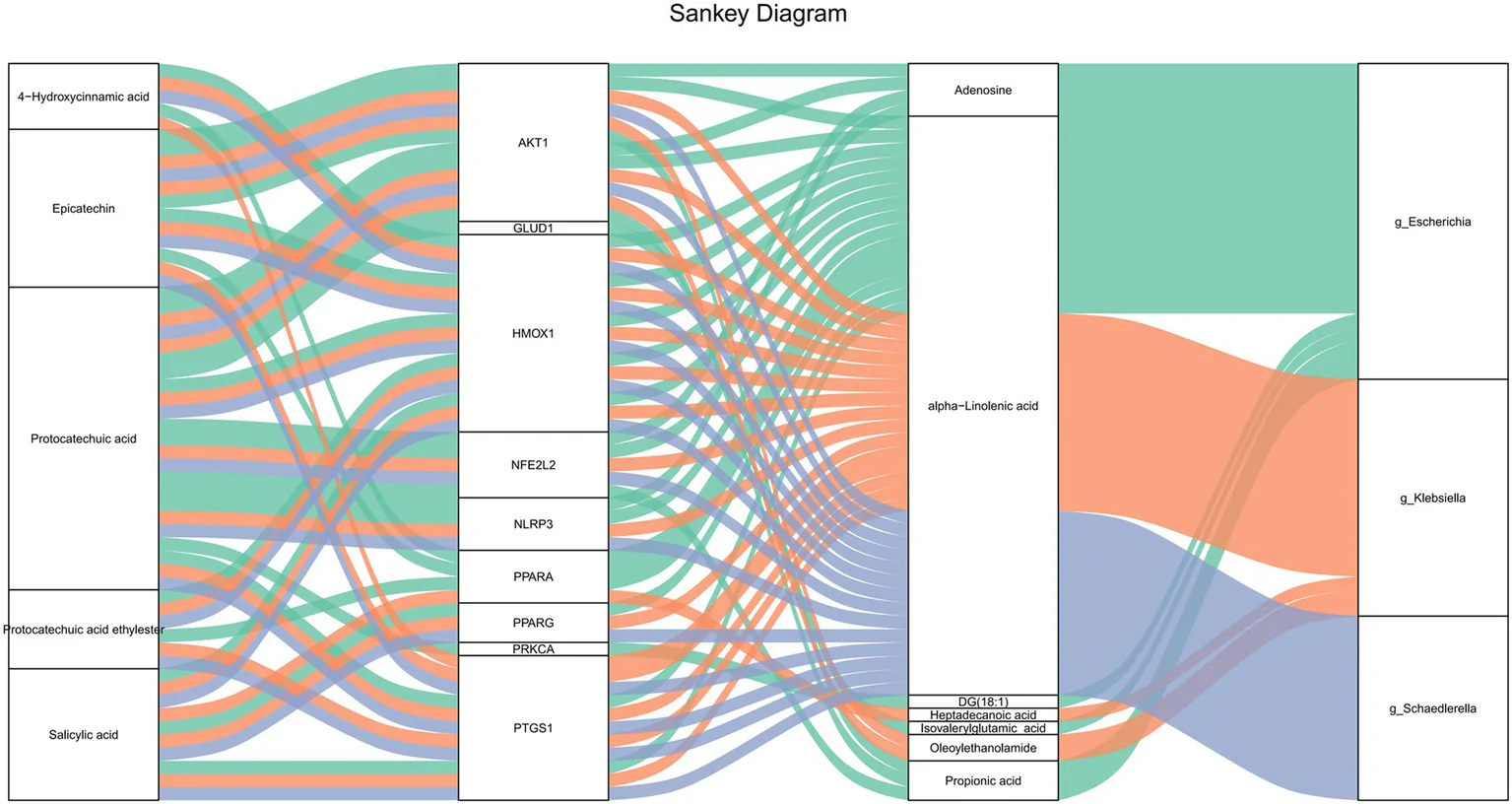
Multilayer Sankey diagram of circulating components–targets–metabolites–gut microbiota.

## 4 Discussion

There is accumulating evidence that supports the important involvement of gut microbiota and its metabolites in the recovery following stroke (Chidambaram et al., 2022; Khan et al., 2024). We focused on whether the neuroprotective effects of XDD against CIRI are linked to its modulation of the gut microbiome and metabolite landscape. Guided by the detection of XDD components entering the blood circulation, network pharmacology research found that the molecular targets of XDD’s effects on cerebral ischemia–reperfusion injury mainly focused on the biological processes such as lipid metabolism, inflammatory cascade reaction and externally triggered cell death pathway. These pathways substantially overlapped with key pathways enriched by differential metabolites in our metabolomics analysis, converging on processes governing inflammation and energy metabolism. These findings support a mechanistic framework in which a “gut microecology–metabolic reprogramming–inflammatory signaling” axis may represent a principal route through which XDD provides protection in CIRI (Figure 12). Consistent with this, XDD improved neurological and cognitive performance, reduced infarct volume, decreased blood viscosity, and attenuated neuronal injury in rats with CIRI.

**Figure 12:**
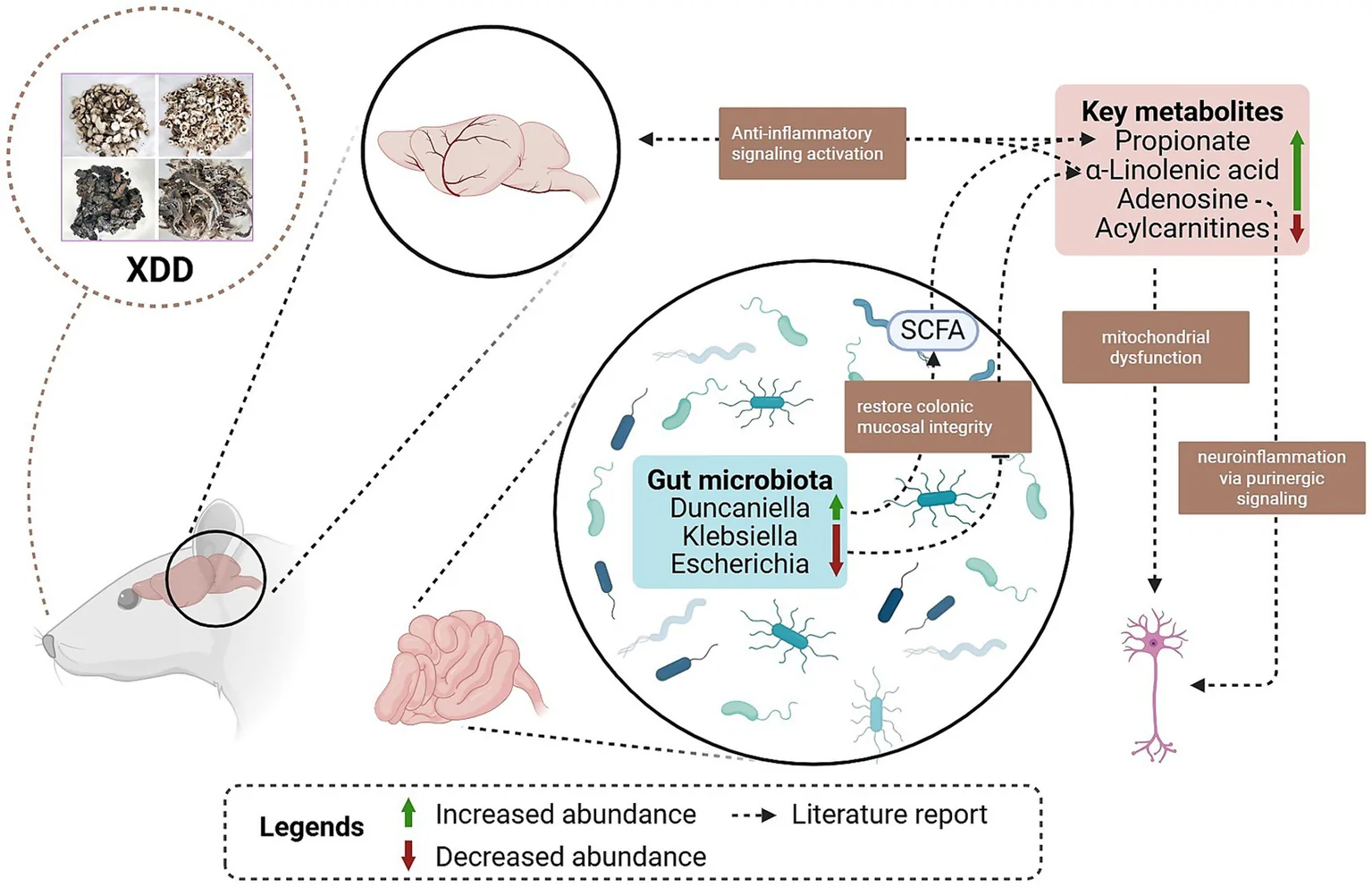
Proposed mechanism of XDD against cerebral ischemia–reperfusion injury through gut microbiota and metabolite regulation.

The 16S rDNA sequencing results showed that CIRI disrupted gut microbial community structure, while XDD intervention partially restored it. Analysis of α- and β-diversity demonstrated a substantial decline in microbial diversity in the MCAO/R group in contrast to the sham controls, with XDD intervention enhancing diversity, particularly in the high-dose group. The findings point to XDD’s ability to alleviate the loss of gut microbiota diversity related to CIRI.

At the phylum level, CIRI-induced dysbiosis was marked by a reduction in Firmicutes and an expansion of potentially pathogenic Proteobacteria and Patescibacteria, consistent with previous reports (Zhao L. et al., 2022; Zhang W. et al., 2024). Excessive Proteobacteria proliferation has been proposed as a biomarker of microbiota instability and a potential diagnostic feature of dysbiosis (Shin et al., 2015). It has been reported that Proteobacteria abundance correlates with infarct burden and inflammatory mediators in experimental CIRI (Ding et al., 2022). At the genus level, the MCAO/R group exhibited higher levels of *Escherichia* and lower levels of *Lactobacillus*. *Escherichia* includes opportunistic pathogens that can drive systemic inflammation when translocated, increasing cerebrovascular risk through inflammatory signaling, immune crosstalk, and barrier disruption (Tenaillon et al., 2010). In contrast, *Lactobacillus* is widely recognized as a beneficial genus with immunomodulatory and antioxidant properties (Tang et al., 2017). The phylum- and genus-levelshifts indicate that XDD significantly remodels the dysbiotic gut community in CIRI. The patterns across taxonomic levels suggest functional relevance in maintaining intestinal homeostasis and shaping immune responses.

For example, increased *Allobaculum* may favor short-chain fatty acid production, thereby strengthening intestinal barrier integrity (Chen et al., 2023); enrichment of *Clostridium ramosum* may influence bile acid metabolism, with downstream effects on host energy balance and inflammatory tone (Pedersen et al., 2022). By suppressing harmful taxa like *Phascolarctobacterium*, it may be possible to reduce the liberation of pathogen-associated molecular patterns and mitigate systemic inflammation (Li et al., 2022). These findings suggest that XDD restores microbial diversity and may achieve therapeutic benefit by reshaping specific functional taxa, supporting microbiome-oriented strategies to protect against ischemia–reperfusion injury.

Untargeted metabolomics captured the systemic metabolic impact of CIRI, identifying 970 metabolites that significantly differed between sham and MCAO/R groups in cecal contents, highlighting metabolic remodeling after ischemia–reperfusion. XDD treatment induced a “reverse regulation” pattern in 347 metabolites, indicating a corrective effect on CIRI-associated metabolic disturbances.

KEGG pathway analysis shows that these metabolites are closely related to many biological processes, such as neuroactive ligand-receptor interactions, cAMP signaling cascade, ABC transporter pathways, and glycophorophospholipid metabolic routes. These findings indicate that XDD may mitigate CIRI by coordinating the regulation of lipid metabolism and neuroinflammatory signaling. Among the metabolites with reversal trends, hexadecatrienoylcarnitine (Car(16:3)), adenosine, and α-linolenic acid (ALA) are particularly noteworthy Car(16:3) belongs to the acylcarnitine family and is tightly linked to mitochondrial energy metabolism (Kang et al., 2018). During ischemic stress, especially when glucose availability is limited, cells may increase their reliance on fatty-acid β-oxidation. In this process, acylcarnitines are essential, as they aid in the transport of fatty acids into the mitochondria (Mavroudakis and Lanekoff, 2023). However, accumulation of long-chain acylcarnitines can contribute to mitochondrial dysfunction, weaken astrocytic metabolic support, and exacerbate neuronal injury (Huang et al., 2024). In this study, Car(16:3) levels were lower in both the sham and XDD-H groups than in the MCAO/R group, which supporting a metabolic normalization effect consistent with a reduction in mitochondrial stress.

It should also be noted that the network pharmacology analysis was primarily based on human databases, whereas experimental validation was conducted in rats. Nevertheless, several key targets identified in this study, including AKT1, NLRP3, HMOX1, and PPAR-related signaling molecules, are highly conserved between humans and rodents. Future studies using rat-specific databases and experimental validation are warranted to further improve translational relevance.

Adenosine is a well-established neuroprotective mediator, while ALA serves as an anti-inflammatory essential fatty acid and a precursor for long-chain ω-3 fatty acids, such as eicosapentaenoic acid (EPA) and docosahexaenoic acid (DHA) (Baker et al., 2016). The depletion of adenosine and ALA following CIRI is frequently linked to increased excitotoxicity and heightened inflammatory injury (Zhou et al., 2015). Studies indicate that DHA can alleviate ischemic brain injury through the modulation of pro-inflammatory cytokines (e.g., IL-1β and IL-1Ra) and by downregulating NF-κB–related gene expression (Kotlega et al., 2025). Notably, XDD treatment effectively reversed abnormalities in these metabolites, suggesting that it may counteract CIRI by restoring energy homeostasis, reducing neuroinflammation, and modulating cellular signaling pathways. From a translational perspective, these metabolomic findings offer a contemporary biochemical interpretation of the traditional functions attributed to XDD (e.g., “clearing heat,” “cooling blood,” and “resolving stasis”).

Correlation analyses revealed a significant link between the gut microbiota and the metabolite network, which may represent a key mechanism for XDD-mediated protection. Specifically, *Klebsiella* exhibited significant negative correlations with MG(18:3), α-linolenic acid, and N-acetylglycylglycine. As an opportunistic pathogen, the overgrowth of *Klebsiella* is often associated with barrier impairment and inflammatory states (Dong et al., 2022). Its negative association with protective metabolites suggests that it may exacerbate metabolic dysregulation by depleting or inhibiting the biosynthesis of beneficial lipids such as ALA. In contrast, *Duncaniella* and *Schaedlerella* showed positive correlations with NAE(18:1), MG species, and ALA, suggesting that changes in the abundance of these genera may be associated with the maintenance of protective metabolites. Although direct functional evidence for *Duncaniella* and *Schaedlerella* is limited, their co-variation with beneficial metabolites is partly consistent with patterns reported for commensal taxa such as *Akkermansia*, which has been associated with barrier maintenance and short-chain fatty acid production. For example, previous studies have reported that a member of the *Duncaniella* genus was associated with enhanced colonic barrier integrity and protection against DSS-induced injury (Chang et al., 2021). Zhang J. et al. (2024) also reported that the restoration of colonic mucosal integrity and blood–brain barrier function, as well as a reduction in hippocampal neuronal damage, was closely associated with an increased *Duncaniella* abundance.

Among the metabolites, NAE(18:1) is identified as a lipid signaling molecule that is rapidly synthesized from membrane phospholipids upon stimulation and serves as a potent agonist of peroxisome proliferator-activated receptor-α (PPAR-α). Given the established role of PPAR-α in lipid metabolism and inflammatory regulation, these findings raise the possibility that XDD-associated microbial changes may be linked to lipid signaling and downstream metabolic pathways in CIRI. Furthermore, the observed associations imply that XDD may augment the metabolic flux of α-linolenic acid and related pathways, potentially facilitating the production of anti-inflammatory long-chain ω-3 fatty acids, including EPA and DHA, which may further assist in the resolution of inflammation during CIRI.

Our findings indicate that XDD may have therapeutic effects by reshaping gut microbiota composition—reducing pathogenic taxa (e.g., *Klebsiella*) and promoting beneficial commensals associated with protective metabolites (e.g., *Duncaniella* and *Schaedlerella*). This shift in microbial community structure may be associated with host metabolic reprogramming. For instance, the restoration of ALA and propionate could enhance anti-inflammatory signaling, potentially by suppressing NF-κB–associated pathways, and improve energy metabolism. In contrast, normalizing adenosine levels may reduce neuroinflammation through purinergic signaling. In parallel, shifts in sphingolipid species (e.g., D-erythro-sphingosine C-20) and acylcarnitines (e.g., hexadecatrienoylcarnitine) suggest that XDD may influence mitochondrial fatty-acid oxidation and membrane stability.

In summary, XDD may protect against CIRI by synchronizing gut microbiota remodeling—specifically, inhibiting opportunistic pathogens while promoting beneficial taxa—with host metabolic reprogramming. Key mechanistic pathways include glycerophospholipid metabolism, linoleic/α-linolenic acid metabolism, purine metabolism, and sphingolipid metabolism. For example, an imbalance between DG and PC/PE can compromise membrane integrity and lipoprotein secretion, while restoring adenosine metabolism may help reduce excitotoxicity. This coordinated regulation of pathways may be related to bioactive constituents in XDD, such as flavonoids, triterpenoid saponins, and polysaccharides, which may contribute to gut microbial remodeling.

## 5 Conclusion

This study systematically evaluated the therapeutic effects of Xijiao Dihuang Decoction (XDD) against cerebral ischemia–reperfusion injury (CIRI). By integrating serum pharmacochemistry, network pharmacology, molecular docking, and multi-omics analyses, we showed that XDD-derived circulating components, especially representative phenolic compounds such as epicatechin and protocatechuic acid, may interact with targets related to inflammatory regulation, oxidative stress response, lipid mediator metabolism, and cell-survival signaling, including AKT1, PTGS1, and HMOX1. In the MCAO/R rat model, XDD alleviated neurological deficits, reduced cerebral infarct volume, improved hemorheological abnormalities, attenuated histopathological injury, and lowered circulating inflammatory cytokine levels, indicating clear neuroprotective effects.

Moreover, integrated 16S rDNA sequencing and untargeted metabolomics demonstrated that XDD effectively corrected CIRI-induced gut microbiota dysbiosis and metabolic disturbances. This correction was characterized by the restoration of beneficial taxa, suppression of potentially pathogenic bacteria, and remodeling of lipid- and amino acid–related metabolic pathways. Correlation analyses indicated coordinated changes between specific bacterial genera and various lipid/energy metabolites, which aligned with improvements in inflammatory phenotypes. Taken together, these findings suggest that XDD exerts broad neuroprotective effects against CIRI by coordinating and regulating the “intestinal-brain axis” (it integrates “intestinal microorganisms-lipids/energy metabolism-inflammation/apoptosis signals”).

Nevertheless, several limitations should be acknowledged. First, although XDD altered gut microbiota composition and metabolic profiles, the present study cannot determine whether these changes were a cause or a consequence of neuroprotection. Future studies using antibiotic depletion, microbiota-depleted models, and cecal microbiota transplantation are needed to establish causality. Second, edaravone was used as a positive control to validate the MCAO/R model and benchmark neuroprotective efficacy, but its effects on gut microbiota and metabolic remodeling were not specifically investigated. Third, key proteins predicted by network pharmacology, including AKT/NLRP3-, caspase-1-, and PPAR-related signaling molecules, were not experimentally validated. Therefore, these mechanisms should be interpreted as predictive rather than confirmed, and future studies should include protein-level validation in brain tissue. Fourth, intestinal barrier function was assessed only by H&E staining, without functional permeability assays or tight-junction protein detection. Finally, the sample size was relatively limited, only male rats were included, the observation period was short, and microbiome analysis was mainly performed at the genus level.

## Data Availability

The raw 16S rRNA gene sequencing data generated in this study have been deposited in the NCBI Sequence Read Archive (SRA) under BioProject accession number PRJNA1529314 and are publicly available at https://www.ncbi.nlm.nih.gov/bioproject/PRJNA1529314. The untargeted LC–MS/MS metabolomics data generated in this study have been deposited in the MetaboLights repository under accession number MTBLS15783 and are publicly available at https://www.ebi.ac.uk/metabolights/MTBLS15783.
